# Clinical outcomes and euploidy rates of embryos derived from zygotes with nonpronuclear (0PN) and monopronuclear (1PN) in IVF or ICSI cycles: a systematic review and meta-analysis

**DOI:** 10.1093/humupd/dmag015

**Published:** 2026-06-04

**Authors:** Hoi Wing Chan, Chi Chiu Wang, David Yiu Leung Chan

**Affiliations:** Assisted Reproductive Technology Unit, Department of Obstetrics and Gynaecology, Faculty of Medicine, The Chinese University of Hong Kong, Hong Kong, China; Department of Obstetrics and Gynaecology, The Chinese University of Hong Kong, Hong Kong, China; Assisted Reproductive Technology Unit, Department of Obstetrics and Gynaecology, Faculty of Medicine, The Chinese University of Hong Kong, Hong Kong, China

**Keywords:** assisted reproduction, human, embryo, atypically pronucleated zygotes, embryology

## Abstract

**BACKGROUND:**

During routine fertilization checks, the presence of two pronuclei (2PN) with two polar bodies is generally regarded as normal fertilization. Nonpronuclear (0PN) zygotes often suggest failed fertilization, but some do cleave and develop into viable embryos due to delayed appearances of pronuclei that were originally missed. Monopronuclear (1PN) zygotes may result from a lack of maternal or paternal pronuclei, fusion of pronuclei, or asynchronous appearance of pronuclei. These embryos may sometimes be transferred when no others are available.

**OBJECTIVE AND RATIONALE:**

Current evidence on the euploidy rates (ER) and clinical outcomes of embryos derived from 0PN and 1PN zygotes is discordant across publications, resulting in conflicting conclusions regarding whether they are suitable for transfer. This review aims to present an updated synthesis of current knowledge regarding the potential adverse outcomes associated with 0PN and 1PN embryo transfers and to identify important gaps that should be addressed in future studies.

**SEARCH METHODS:**

CENTRAL, Embase, Fertility and Sterility, MEDLINE, and PubMed databases were searched on 10 November 2025 using the keywords ‘0PN embryo’ and ‘1PN embryo’. All types of studies, except case reports and reviews, were included if they compared 0PN/1PN with 2PN zygotes in women of any age undergoing IVF/ICSI cycles, followed by single embryo transfer (SET) and/or preimplantation genetic testing for aneuploidy (PGT-A). Pronuclear status was assessed 16–18 h post insemination. Only data from SET cycles were included for embryo transfer outcomes.

**OUTCOMES:**

Eleven studies reporting on 37 240 SET cycles and 27 195 biopsied blastocysts were included. Compared to 2PN embryos, those derived from 0PN zygotes showed no significant differences in live birth rate (LBR) (OR = 0.94, 95% CI = 0.83–1.06, *P* = 0.33, *I*^2^ = 2%), clinical pregnancy rate (CPR) (OR = 0.96, 95% CI = 0.85–1.08, *P* = 0.48, *I*^2^ = 0%), miscarriage rate (MR) (OR = 1.04, 95% CI = 0.84–1.29, *P* = 0.70, *I*^2^ = 0%), malformation rate (OR = 1.05, 95% CI = 0.45–2.45, *P* = 0.91, *I*^2^ = 0%), and ER (OR = 0.86, 95% CI = 0.50–1.48, *P* = 0.59, *I*^2^=63%). However 0PN-derived embryos had a significantly lower blastulation rate (OR = 0.34, 95% CI = 0.17–0.68, *P* = 0.002; *I*^2^ = 99%). Compared to 2PN, 1PN embryos showed a significantly lower LBR (OR = 0.83, 95% CI = 0.70–0.98, *P* = 0.03, *I*^2^ = 12%), CPR (OR = 0.81, 95% CI = 0.68–0.96, *P* = 0.01, *I*^2^ = 22%), and blastulation rate (OR = 0.26, 95% CI = 0.14–0.48, *P* < 0.001; *I*^2^ = 96%), but a similar MR (OR = 1.34, 95% CI = 0.76–2.37, *P* = 0.31, *I*^2^ = 67%), malformation rate (OR = 0.46, 95% CI = 0.16–1.32, *P* = 0.15, *I*^2^ = 0%), and ER (OR = 1.05, 95% CI = 0.70–1.58, *P* = 0.80, *I*^2^ = 66%).

**WIDER IMPLICATIONS:**

In the meta-analysis, both 0PN- and 1PN-derived embryos showed no significant differences in rates of euploidy, miscarriage, and malformations compared to 2PN-derived embryos. However, clinical pregnancy and LBRs were significantly lower for 1PN, but not 0PN, embryos than for 2PN embryos. These findings suggest that 0PN-, and possibly even 1PN-, derived embryos, especially those that have undergone PGT-A, may represent a viable source for embryo transfer in certain clinical settings, particularly in cases where 2PN embryos are limited or unavailable.

**REGISTRATION NUMBER:**

CRD42024546685.

## Graphical abstract

**Figure:**
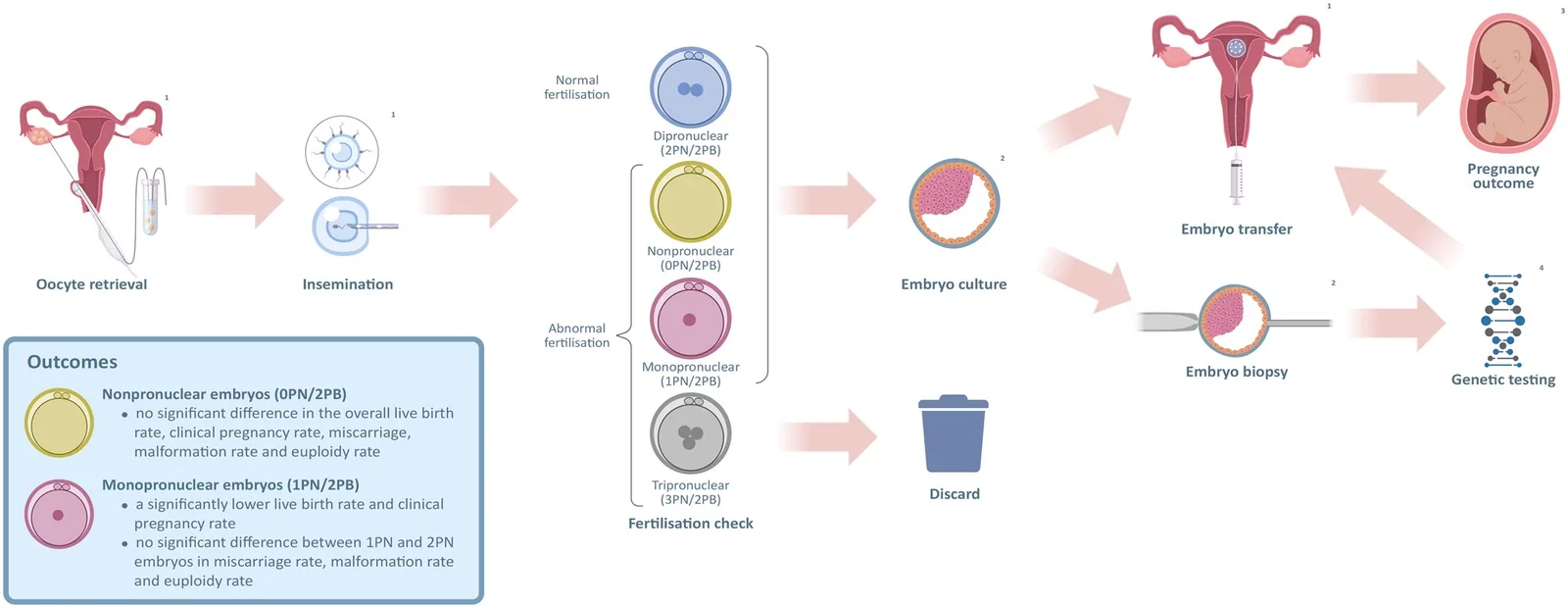
The process from oocyte retrieval to embryo transfer, summarizing the key finds of the review.

## Introduction

In human ART treatment, normal fertilization is generally considered by the appearance of two pronuclei (2PN) and two polar bodies (2PB) at the time of fertilization assessment, usually 16–18 h post-insemination (Balaban *et al.*, 2011; ESHRE Guideline Group on Good Practice in IVF Labs *et al.*, 2016). For fertilization, a sperm penetrates the zona pellucida and fuses with the oolemma. The activated oocyte resumes meiosis and extrudes the second polar body (PB). It takes ∼2–3 h from the moment a sperm penetrates the zona pellucida to the extrusion of the second PB (Nagy *et al.*, 1994). The first pronucleus (PN) forms 4–5 h afterwards, and the second PN forms simultaneously or shortly after, in <10 min in most oocytes (Nagy *et al.*, 1994; Payne *et al.*, 1997).

For zygotes that are not derived from the typical 2PN configuration, the term atypically pronucleated zygotes (APZs) was first introduced by Capalbo *et al.* (2024). This term is used to describe zygotes that do not exhibit the normal 2PN morphology. The most frequently observed APZ is a tripronuclear (3PN) zygote, which could be a result of multiple sperm penetrations, fertilization by a diploid sperm, or an oocyte failing on first meiotic division, and is characterized by the appearance of 3PN and 2PB (Rosenbusch, 2008). In the case of 3PN and 1PB, the oocyte fails to extrude the second PB, forming two female PN (Rosenbusch, 2008). The 3PN zygotes have been reported to have a triploidy rate of 43.3 and 74.1% by Mutia *et al.* (2019) and Girardi *et al.* (2024), respectively, and are usually discarded by the laboratory. Therefore, they were not included in this meta-analysis.

Other APZs include nonpronuclear (0PN) and monopronuclear (1PN) zygotes. While the observation of 0PN zygote most often suggests failed fertilization, some do cleave and develop into viable embryos. This is likely due to the early fading or late appearance of pronuclei; therefore, the appearance of no pronuclei was observed during fertilization assessment (Liu *et al.*, 2016). Meanwhile, there are several hypotheses on the formation of 1PN: (i) the 1PN zygote could be a result of a lack of maternal or paternal PN, resulting in a haploid cell; (ii) it could sometimes be the result of the fusion of maternal and paternal pronuclei; or (iii) the appearance of pronuclei occurs asynchronously, yet resulting in a diploid zygote (Evans *et al.*, 2019). Time-lapse analysis has shown that when a sperm fuses with the oolemma within 18 μm of the site of second PB extrusion, there is a high chance of a single PN forming due to the close proximity of the paternal and maternal chromosomes (Wei *et al.*, 2022). However, this scenario is largely avoided in ICSI, as the site of sperm entry is controlled, thereby reducing the likelihood that paternal and maternal chromosomes will be enclosed within a single PN.

Embryos derived from zygotes where 2PN was not observed are sometimes transferred when patients have no 2PN embryos available for transfer. Reports of successful live births following such transfers (Gras and Trounson, 1999; Burney *et al.*, 2008; Itoi *et al.*, 2015; Liu *et al.*, 2016) have led to studies investigating the clinical outcomes after the transfer of such embryos as well as their genetic profiles. Euploidy rates range from as little as 3% to upwards of 75% for 0PN embryos (Noyes *et al.*, 2008; Kayisli *et al.*, 2010; Catala *et al.*, 2017; Destouni *et al.*, 2018; McCaffrey *et al.*; 2019a; Tong *et al.*, 2023) and 5–72% for 1PN embryos (Sarajari *et al.*, 2005; Noyes *et al.*, 2008; Liao *et al.*, 2009; Bradley *et al.*, 2017; Capalbo *et al.*, 2017b; Xie *et al.*, 2018; Hirata *et al.*, 2020; Wozniak *et al.*, 2021; Ussher *et al.*, 2023). A recent review reported that among 0PN embryos, 95.1% were diploid, 3.7% haploid, and 1.2% polyploid, while among 1PN embryos, 70.5% were diploid, 27.2% haploid, and 2.2% polyploid (Capalbo *et al.*, 2024). In comparison, 2PN embryos were found to be 98.9% diploid, with only 0.2% being haploid and 0.9% triploid (Picchetta *et al.*, 2023).

Notably, recent high-resolution genetic analyses have suggested that the developmental competence and chromosomal constitution of 1PN embryos may also vary according to the insemination method. Xiong *et al.* (2024) reported that 1PN blastocysts in ICSI cycles had a significantly lower diploid euploidy rate compared with those in IVF cycles (25.00% versus 75.44%, *P* = 0.001), and all four uniparental diploid embryos identified using single nucleotide polymorphism analysis originated from ICSI-derived 1PN zygotes. These findings indicate that the mechanisms underlying 1PN formation may differ between IVF and ICSI, with important implications for the chromosomal constitution of resulting embryos.

There have also been reports of healthy live births resulting from the transfer of 0PN and 1PN embryos without preimplantation genetic testing for aneuploidy (PGT-A) done prior to the transfer. The live birth rate (LBR) ranges from 26 to 48% for 0PN embryos (Hondo *et al.*, 2019; Li *et al.*, 2021; Chen *et al.*, 2022; Zhu *et al.*, 2024a) and 19 to 27% for 1PN embryos (Hondo *et al.*, 2019; Si *et al.*, 2019; Li *et al.*, 2021). However, according to ESHRE Guideline Group on Good Practice in IVF Labs et al. (2016), it is not recommended to use 0PN or 1PN embryos, even when no 2PN embryos are available for transfer. Zygotes, where 2PN is not observed, are often routinely discarded by many laboratories. Yet 0PN and 1PN embryos could be of value to patients with various medical conditions to generate embryos through subsequent ART treatment. This review synthesises current evidence on the clinical outcomes and euploidy rates of 0PN and 1PN embryos, with the aim of providing insights into their potential utility in ART.

## Methods

This study was performed according to the Cochrane handbook for systematic reviews of interventions (Higgins *et al.*, 2023) and reported according to the Preferred Reporting Items for Systematic Reviews and Meta-Analyses (PRISMA) 2020 statement (Page *et al.*, 2021). The review protocol was registered under the PROSPERO database (CRD42024546685).

### Search strategies

Cochrane Central Register of Controlled Studies (CENTRAL), Embase, Fertility and Sterility, MEDLINE, and PubMed databases were searched on 10 November 2024 using keywords ‘0PN’ and ‘1PN’ without restrictions on publication date and language (see Supplementary Data File S1).

European Society of Human Reproduction & Embryology (ESHRE) 30th to 36th annual meeting abstracts were hand-searched.

### Eligibility criteria and study selection

There were no randomized controlled trials available, so all other types of studies, except case reports and reviews, were included if they involved women of any age who underwent IVF or ICSI cycles followed by single embryo transfer (SET) and/or trophectoderm biopsy for PGT-A. Studies involving double embryo transfers were excluded if SET outcomes were not reported separately; when SET outcomes were available, only those were included in the analysis. Fertilization of embryos was determined by pronuclear observation between 16 and 18 h after insemination (Balaban *et al.*, 2011). Studies where pronuclear observation was not specified or outside of the above time frame were excluded.

Titles and abstracts of studies retrieved from database searches were first scanned to remove those that were irrelevant to the topic. The articles were then examined to see whether they met the inclusion criteria. Characteristics of included studies are detailed in Supplementary Data File S2. Reasons for exclusion for each excluded study are detailed in the Supplementary Table S1.

### Data extraction and quality assessment

Data extracted from different studies were combined into a data extraction form. The following were the study characteristics included in the data extraction form.

For study design, study type, date of the study, duration of study, and sampling method were included. For participants, age range, mean age and SD, inclusion and exclusion criteria, and indication for IVF and/or PGT-A were included. For eligibility, time of fertilization assessment and number of embryos transferred per cycle were included. For methods, the method of insemination, cleavage-stage or blastocyst-stage embryo transfer, fresh or frozen embryo transfer, PGT-A methods, and culturing incubators were included. For interventions, embryo transfer or biopsy of 0PN or 1PN embryos was included. For outcomes, LBR (defined as the number of live births per embryo transferred), clinical pregnancy rate (CPR) (defined as the number of clinical pregnancies per embryo transferred), miscarriage rate (defined as the number of miscarriages per clinical pregnancy), malformation rate (defined as the number of malformations per live birth), blastulation rate (defined as the number of blastocysts formed per fertilized zygote), euploidy rate (defined as the number of euploid embryos per embryo biopsied), and the rate of bi-parental inheritance (defined as the number of euploid embryos with chromosomes inherited from both parents per embryo biopsied) were included.

Since most of the included studies were non-randomized observational studies, the Risk Of Bias In Non-randomized Studies—of Exposures (ROBINS-E) tool was used to assess the risk of bias of each study. Each study was assessed based on the bias due to confounding, bias arising from measurement of the exposure, bias in selection of participants into the study, bias due to post-exposure interventions, bias due to missing data, bias in measurement of the outcome, and bias in selection of the reported result, as described in the ROBINS-E tool manual (Higgins *et al.*, 2024). The overall risk-of-bias judgement for each study can be classified as ‘low risk’, ‘some concerns’, ‘high risk’, or ‘very high risk’.

### Data analysis

Dichotomous results were expressed as odds ratios (ORs) with 95% CIs using the Mantel–Haenszel method, with the exception of rare events (i.e. malformation rate), where the Peto method was used.

Heterogeneity between results from different studies was assessed using the *I*^2^ statistic, which was then interpreted according to the guidelines from the Cochrane Handbook for Systematic Reviews of Interventions (Higgins *et al.*, 2023): 0–40%: might not be important; 30–60%: moderate heterogeneity; 50–90%: substantial heterogeneity; 75–100%: considerable heterogeneity.

Subgroup analyses were performed on the methods of insemination (IVF versus ICSI) for all outcomes with sufficient data to assess the effect on heterogeneity. Sensitivity analyses were conducted using the number of embryos transferred as the denominator for embryo-transfer outcomes (miscarriage and malformation rates), in place of the original denominators. The meta-analysis was done using Review Manager (RevMan) [Computer program] version 7.2.0 (The Cochrane Collaboration, 2024).

Reporting biases were investigated using funnel plots and Egger’s test (Egger *et al.*, 1997), generated with Comprehensive Meta-Analysis Version 4 (Borenstein et al., 2022). Intercepts with 95% CIs were presented, and evidence of asymmetry was based on a one-tailed *P*-value of <0.1.

### Certainty assessment

Summary of findings tables were generated using GRADEpro GDT (2024), which evaluated the overall quality of the body of evidence for LBR, CPR, miscarriage rate, malformation rate, blastulation rate, and euploidy rate, using the GRADE criteria, i.e. risk of bias, inconsistency, imprecision, indirectness, and publication bias (Higgins *et al.*, 2023). Reasons for downgrading the certainty of the evidence were listed in the explanations section under the tables.

## Results

### Literature search

A total of 1123 studies were identified through electronic searches. After 607 duplicates were removed, 516 studies were screened, and 417 studies were excluded at title and abstract screening. After assessment for eligibility, 1 of the remaining 99 studies was not retrieved, and 87 studies were excluded with reasons detailed in Fig. 1 and Supplementary Table S1. A total of 11 eligible studies were included in this review.

**Figure 1.**
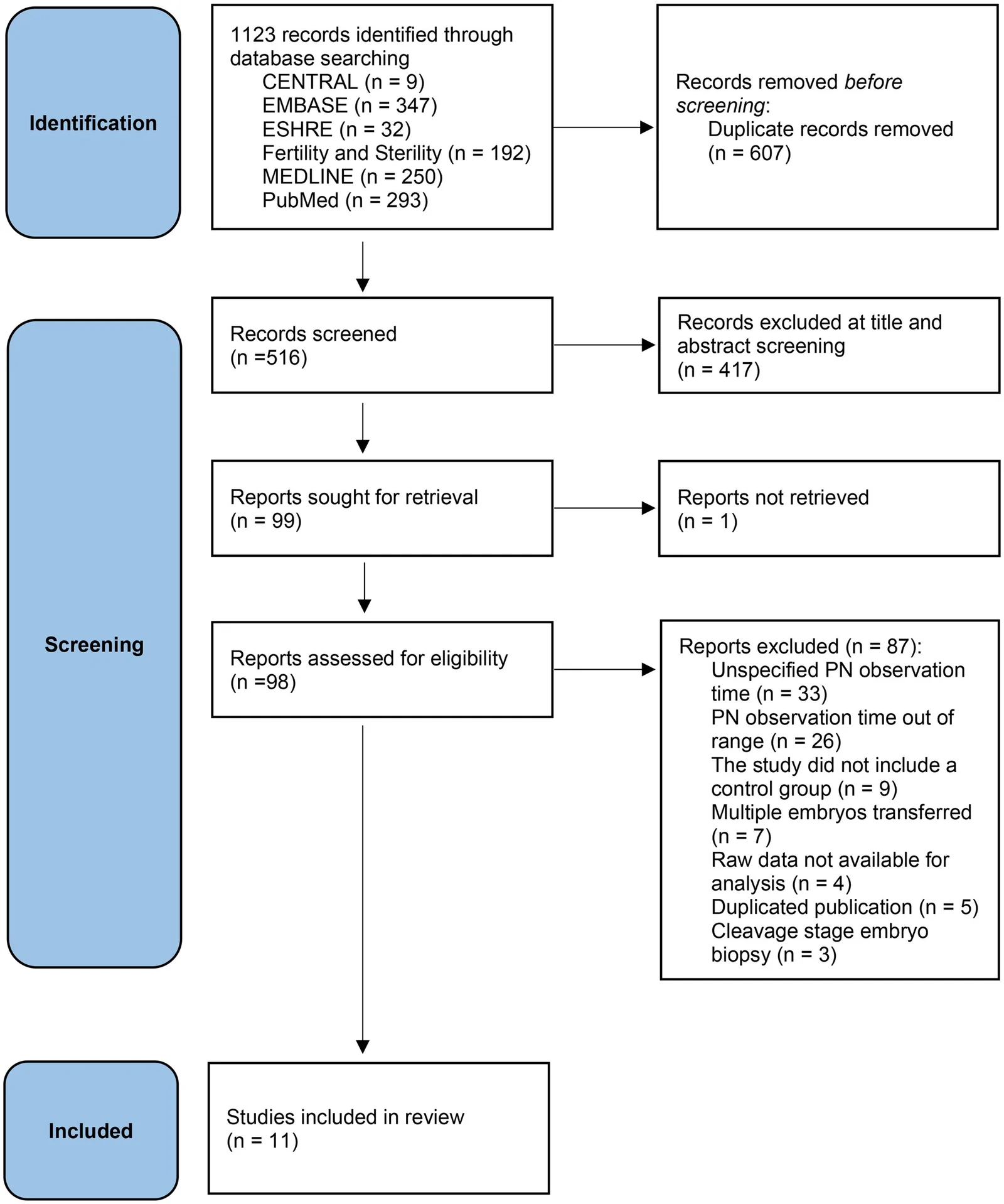
Study flow diagram. PN, pronucleus.

### Study characteristics

Study characteristics are summarized in Table 1 and detailed in Supplementary Data File S2. Overall results are summarized in Table 2.

**Table 1.** Summary of characteristics of included studies.

Author (publication year)	Country	Number of centres	Study design	Study group	Insemination method	Stage of embryos transferred/biopsied	Fresh/frozen embryo transfer	Outcome	ROBINS-E judgement
Bradley *et al.* (2017)	Australia	Multi-centre	Retrospective case–control	1PN	IVF/ICSI	Blastocyst stage	Frozen	Clinical pregnancy rate, euploidy rate, rate of bi-parental inheritance of 1PN embryos	High risk
Chen *et al.* (2020)	China	Single centre	Retrospective case–control	0PN and 1PN	IVF/ICSI	Blastocyst stage	Fresh	Live birth rate, clinical pregnancy rate, miscarriage rate, blastulation rate	High risk
Fu *et al.* (2021)	China	Single centre	Retrospective case–control	0PN	IVF	Blastocyst stage	Frozen	Live birth rate, miscarriage rate, malformation rate, blastulation rate	Some concerns
Hondo *et al.* (2019)	Japan	Single centre	Retrospective case–control	0PN and 1PN	IVF/ICSI	Blastocyst stage	Frozen	Live birth rate, clinical pregnancy rate, miscarriage rate, malformation rate	High risk
Labied *et al.* (2024)	Belgium	Single centre	Retrospective case–control	1PN	IVF/ICSI	Cleavage stage/blastocyst stage	Fresh/frozen	Live birth rate, clinical pregnancy rate, miscarriage rate	Some concerns
Li *et al.* (2021)	China	Single centre	Retrospective case–control	0PN and 1PN	IVF/ICSI IVF only for 1PN	Blastocyst stage	Frozen	Live birth rate, clinical pregnancy rate, miscarriage rate, malformation rate	Some concerns
McCaffrey *et al.* (2019a)	USA	Single centre	Retrospective case–control	0PN and 1PN	IVF/ICSI	Blastocyst stage		Euploidy rate	High risk
Tong *et al.* (2023)	China	Single centre	Retrospective case–control	0PN and 1PN	IVF/ICSI	Blastocyst stage	Frozen	Live birth rate, clinical pregnancy rate, miscarriage rate, malformation rate, blastulation rate, euploidy rate, rate of bi-parental inheritance of 1PN embryos	High risk
Wozniak *et al.* (2021)	USA	Single centre	Retrospective case–control	0PN and 1PN	ICSI	Blastocyst stage		Blastulation rate, euploidy rate, rate of bi-parental inheritance of 1PN embryos	High risk
Xu *et al.* (2025)	China	Single centre	Retrospective case–control	0PN and 1PN	IVF/ICSI IVF only for 1PN	Blastocyst stage	Frozen	Live birth rate, clinical pregnancy rate, miscarriage rate, malformation rate, blastulation rate	Some concerns
Zhu *et al.* (2024b)	China	Single centre	Retrospective case–control	0PN and 1PN	IVF	Blastocyst stage	Frozen	Live birth rate, clinical pregnancy rate, miscarriage rate, malformation rate	High risk

0PN, nonpronuclear; 1PN, monopronuclear; ROBINS-E, Risk Of Bias In Non-randomized Studies—of Exposures.

**Table 2.** Summary of findings table for nonpronuclear (0PN) embryos versus dipronuclear (2PN) embryos and monopronuclear (1PN) embryos versus dipronuclear (2PN) embryos.

0PN embryos compared to 2PN embryos for embryo transfer						
Intervention: 0PN embryos Comparison: 2PN embryos						
Outcome	No of studies	No of patients		Effect		Certainty
		0PN embryos	2PN embryos	Relative (95% CI)	Absolute (95% CI)	
Live birth rate	7	451/1289	11 535/33 559 (34.4%)	OR 0.94	14 fewer per 1000	⨁⨁⨁◯
		(35.0%)		(0.83–1.06)	(from 41 fewer to 13 more)	Moderate^a^
Clinical pregnancy rate	6	569/1258 (45.2%)	14 959/33 290 (44.9%)	OR 0.96	10 fewer per 1000	⨁⨁⨁◯
				(0.85–1.08)	(from 40 fewer to 19 more)	Moderate^a^
Miscarriage rate	6	115/569 (20.2%)	3340/14 959 (22.3%)	OR 1.04	7 more per 1000	⨁⨁◯◯
				(0.84 to 1.29)	(from 29 fewer to 47 more)	Low^a,c^
Malformation rate	6	6/433	148/11 397 (1.3%)	Peto OR 1.05	1 more per 1000	⨁◯◯◯
		(1.4%)		(0.45 to 2.45)	(from 7 fewer to 18 more)	Very low^a,c,d^
Blastulation rate	4	3888/9141	46 161/90 284	OR 0.34	249 fewer per 1000	⨁◯◯◯
		(42.5%)	(51.1%)	(0.17 to 0.68)	(from 360 fewer to 96 fewer)	Very low^a,b,d^
Euploidy rate	3	91/255	7981/21 454 (37.2%)	OR 0.86	35 fewer per 1000	⨁◯◯◯
		(35.7%)		(0.50 to 1.48)	(from 144 fewer to 95 more)	Very low^a,b,c^

1PN embryos compared to 2PN embryos for embryo transfer						
Intervention: 1PN embryos Comparison: 2PN embryos						
Outcome	No of studies	No of patients		Effect		Certainty
		0PN embryos	2PN embryos	Relative (95% CI)	Absolute (95% CI)	
Live birth rate	7	282/1046 (27.0%)	11 470/33 538	OR 0.83	41 fewer per 1000	⨁⨁◯◯
			(34.2%)	(0.70–0.98)	(from 75 fewer to 5 fewer)	Low^a^^,c^
Clinical pregnancy rate	8	395/1072 (36.8%)	15 597/34 610 (45.1%)	OR 0.81	51 fewer per 1000	⨁⨁⨁◯
				(0.68–0.96)	(from 93 fewer to 10 fewer)	Moderate^a^
Miscarriage rate	7	91/386	3344/15 024 (22.3%)	OR 1.34	55 more per 1000	⨁◯◯◯
		(23.6%)		(0.76–2.37)	(from 44 fewer to 182 more)	Very low^a,b,c^
Malformation rate	5	1/245	148/11 298	OR 0.46	7 fewer per 1000	⨁◯◯◯
		(0.4%)	(1.3%)	(0.16–1.32)	(from 11 fewer to 4 more)	Very low^a,b,c,d^
Blastulation rate	3	716/2941 (24.3%)	42 539/84 575 (50.3%)	OR 0.26	295 fewer per 1000	⨁◯◯◯
				(0.14–0.48)	(from 379 fewer to 176 fewer)	Very low^a,b,d^
Euploidy rate	4	237/662 (35.8%)	10 789/26 278 (41.1%)	OR 1.05	12 more per 1000	⨁◯◯◯
				(0.70–1.58)	(from 83 fewer to 113 more)	Very low^a,b,c^

OR: odds ratio.

a Downgraded once for potential risk of bias.

b Downgraded once for substantial heterogeneity.

c Downgraded once for imprecision—small number of events.

d Downgraded once for imprecision—wide confidence interval.

Of the 11 studies included, 9 reported clinical outcomes after SET (Bradley *et al.*, 2017; Hondo *et al.*, 2019; Chen *et al.*, 2020; Fu *et al.*, 2021; Li *et al.*, 2021; Tong *et al.*, 2023; Labied *et al.*, 2024; Zhu *et al.*, 2024b; Xu *et al.*, 2025), among which 7 reported outcomes of transferring 0PN embryos (Hondo *et al.*, 2019; Chen *et al.*, 2020; Fu *et al.*, 2021; Li *et al.*, 2021; Tong *et al.*, 2023; Zhu *et al.*, 2024b; Xu *et al.*, 2025), and seven reported outcomes of transferring 1PN embryos (Bradley *et al.*, 2017; Hondo *et al.*, 2019; Chen *et al.*, 2020; Li *et al.*, 2021; Tong *et al.*, 2023; Labied *et al.*, 2024; Zhu *et al.*, 2024b).

Four of the included studies performed PGT-A and reported the embryo euploidy rate (Bradley *et al.*, 2017; McCaffrey *et al.*, 2019a; Wozniak *et al.*, 2021; Tong *et al.*, 2023), among which three reported the 0PN euploidy rate (McCaffrey *et al.*, 2019a; Wozniak *et al.*, 2021; Tong *et al.*, 2023), and four reported the 1PN euploidy rate (Bradley *et al.*, 2017; McCaffrey *et al.*, 2019a; Wozniak *et al.*, 2021; Tong *et al.*, 2023), including three which reported bi-parental inheritance confirmation for the 1PN embryo (Bradley *et al.*, 2017; Wozniak *et al.*, 2021; Tong *et al.*, 2023).

There were 87 studies excluded (see Supplementary Table S1). Five studies included duplicated data, as the studies were carried out in the same centres as those included with overlapping time periods (Si *et al.*, 2019; McCaffrey *et al.*, 2019b; Wozniak and Schiewe, 2020; Chen *et al.*, 2022; Wang *et al.*, 2022), while 33 studies did not specify PN observation time for fertilization checks (Staessen and Van Steirteghem, 1997; Werlin, 2007; Mizuta *et al.*, 2011; Kim *et al.*, 2013; Tan *et al.*, 2014; Xu *et al.*, 2015; Tsuji *et al.*, 2016; Mateo *et al.*, 2017; Potts *et al.*, 2017; Shimizu *et al.*, 2017; Mizuta *et al.*, 2018; Nohales *et al.*, 2018; Grkovic *et al.*, 2019; Mizuta *et al.*, 2019; Romanelli *et al.* 2019; Tsuji *et al.*, 2020; Brinkmann *et al.*, 2021; Henry *et al.*, 2021; Feferkorn *et al.*, 2022; Jordan *et al.*, 2022; Zhao *et al.*, 2022; Cagnin *et al.*, 2023; Girardi *et al.*, 2023; Perry *et al.*, 2023; Seckin *et al.*, 2023; Shun *et al.*, 2023; Yelke *et al.*, 2023; Henry *et al.*, 2023a,b; Al Hashimi *et al.*, 2024; Wen *et al.*, 2024; Yeshua *et al.*, 2024; Girardi *et al.*, 2025). In 26 studies, the PN observation time was out of the range of 16–18 h post-insemination (Otsu *et al.*, 2004; Campos *et al.*, 2007; Sayers *et al.*, 2011; Heath *et al.*, 2012; Lee *et al.*, 2013; Mateo *et al.*, 2013; Li *et al.*, 2015a,b; Liu *et al.*, 2016; Yao *et al.*, 2016; Destouni *et al.*, 2018; Kai *et al.*, 2018; Picou *et al.*, 2019; Freitas *et al.*, 2020; Hirata *et al.*, 2020; Li *et al.*, 2020; Paz *et al.*, 2020; Kobayashi *et al.*, 2021; Soler *et al.*, 2021; Gomez Peña *et al.*, 2022; Curnow *et al.*, 2023; Zhang *et al.*, 2023; Xiong *et al.*, 2024; Zhu *et al.*, 2024a; Moon *et al.*, 2025; Wang *et al.*, 2025). Nine studies did not include a control group (Noyes *et al.*, 2007; Liao *et al.*, 2009; Catala *et al.*, 2017; Capalbo *et al.*, 2017a,b; Xie *et al.*, 2018; Ussher *et al.*, 2023; McCallie *et al.*, 2024; Stock-Myer *et al.*, 2025). In seven studies, multiple embryo transfers were carried out (Aoi *et al.*, 2010; Itoi *et al.*, 2015; Ma *et al.*, 2018; Wei *et al.*, 2019; Zhang *et al.*, 2019; Rao *et al.*, 2020; Fu *et al.*, 2022). In four studies, raw data were not available for analysis (Al Hashimi *et al.*, 2025, Barberi *et al.*, 2025; Paun *et al.*, 2025; Perry *et al.*, 2025), and in three studies, blastomere biopsy instead of trophectoderm biopsy was carried out (Noyes *et al.*, 2008; Kayisli *et al.*, 2010; Lebovitz *et al.*, 2024).

### Risk of bias assessment

The scoring of each included study was detailed in the table of ‘Quality assessment of studies using the Risk Of Bias In Non-randomized Studies—of Exposure (ROBINS-E) tool’ (Table 3).

**Table 3.** Quality assessment of studies using the Risk Of Bias In Non-randomized Studies—of Exposure (ROBINS-E) tool.

	Bias due to confounding	Bias arising from measurement of the exposure	Bias in selection of participants into the study	Bias due to post-exposure interventions	Bias due to missing data	Bias in measurement of the outcome	Bias in selection of the reported result	Overall
Bradley *et al.* (2017)	High risk	Some concerns	Low risk	Some concerns	Low risk	Low risk	Low risk	High risk
Chen *et al.* (2020)	Very high risk	Some concerns	Low risk	Low risk	Low risk	Low risk	Low risk	High risk
Fu *et al.* (2021)	Some concerns	Some concerns	Low risk	Some concerns	No information	Some concerns	Low risk	Some concerns
Hondo *et al.* (2019)	High risk	Some concerns	Low risk	Very high risk	No information	High risk	Low risk	High risk
Labied *et al.* ( 2024)	Low risk	Some concerns	High risk	Low risk	No information	Low risk	Low risk	Some concerns
Li *et al.* (2021)	Some concerns	Some concerns	High risk	No information	Low risk	Some concerns	Low risk	Some concerns
McCaffrey *et al.* (2019a)	High risk	Some concerns	No information	Some concerns	No information	No information	Low risk	High risk
Tong *et al.* (2023)	Very high risk	Some concerns	Low risk	Some concerns	Some concerns	Some concerns	Low risk	High risk
Wozniak *et al.* (2021)	Very high risk	Some concerns	No information	No information	No information	Some concerns	Low risk	High risk
Xu *et al.* (2025)	Low risk	Some concerns	High risk	No information	Some concerns	Some concerns	Low risk	Some concerns
Zhu *et al.* (2024b)	High risk	Some concerns	Low risk	High risk	Some concerns	Some concerns	Low risk	High risk

Four of the included studies were classified as having ‘some concerns’ (Fu *et al.*, 2021; Li *et al.*, 2021; Labied *et al.*, 2024; Xu *et al.*, 2025). Reasons for such judgement were possible bias from measurement of the exposure due to the limitation of single time-point assessment and bias due to post-exposure interventions such as different culture conditions, exclusion of cycles, and re-checking of the number of pronuclei.

Seven of the included studies were classified as ‘high risk’ (Bradley *et al.*, 2017; Hondo *et al.*, 2019; McCaffrey *et al.*, 2019a; Chen *et al.*, 2020; Wozniak *et al.*, 2021; Tong *et al.*, 2023; Zhu *et al.*, 2024b). Reasons for such judgement other than those mentioned above were bias due to confounding and (understandably) prioritization of 2PN embryo transfers. Confounding by maternal age is particularly relevant for euploidy outcomes, given the well-established association between maternal age and aneuploidy. However, none of the included studies reporting euploidy rates adjusted for maternal age (Bradley *et al.*, 2017; McCaffrey *et al.*, 2019a; Wozniak *et al.*, 2021; Tong *et al.*, 2023).

### Outcome comparison: 0PN embryos versus 2PN embryos

#### Live birth rate

Seven of the 11 included studies reported LBRs after transferring 0PN embryos (Hondo *et al.*, 2019; Chen *et al.*, 2020; Fu *et al.*, 2021; Li *et al.*, 2021; Tong *et al.*, 2023; Zhu *et al.*, 2024b; Xu *et al.*, 2025), and there was no significant difference between 0PN and 2PN embryos on the LBR (Fig. 2a; OR = 0.94, 95% CI = 0.83–1.06, *P*-value = 0.33; seven studies, N = 34 848; *I*^2^ = 2%). Both the asymmetry of the funnel plot (Supplementary Fig. S1a) and the Egger’s test (intercept (*Y*) = −1.01, 95% CI = −2.63 to 0.60, *t* = 1.61, one-tailed *P*-value = 0.08) indicate possible publication bias.

**Figure 2.**
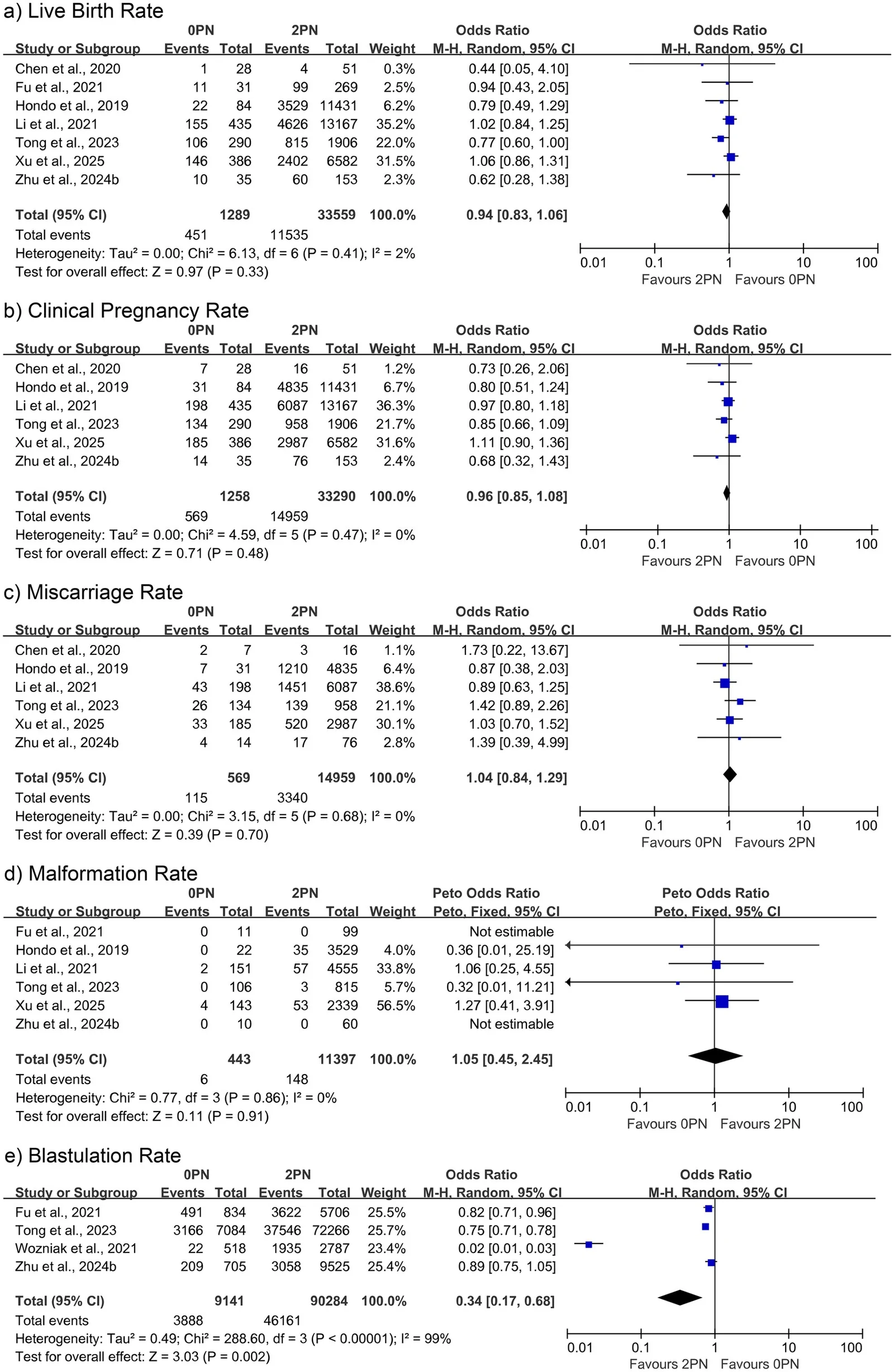
Comparison of outcomes between nonpronuclear (0PN) and dipronuclear (2PN) embryos. There were no significant differences in live birth rate, clinical pregnancy rate, miscarriage rate, malformation rate, or euploidy rate between 0PN and 2PN embryos; however, 0PN embryos had a significantly lower blastulation rate compared with 2PN embryos. Forest plots of (a) live birth rate, (b) clinical pregnancy rate, (c) miscarriage rate, (d) malformation rate, (e) blastulation rate, and (f) euploidy rate between 0PN embryos and 2PN embryos. Each plot presents the odds ratios (ORs) with 95% CIs for individual studies, with pooled estimates calculated using a random-effects model. Horizontal lines represent individual study estimates, and diamonds represent overall effects. Heterogeneity was assessed using the I2 statistic.

**Figure 2.**
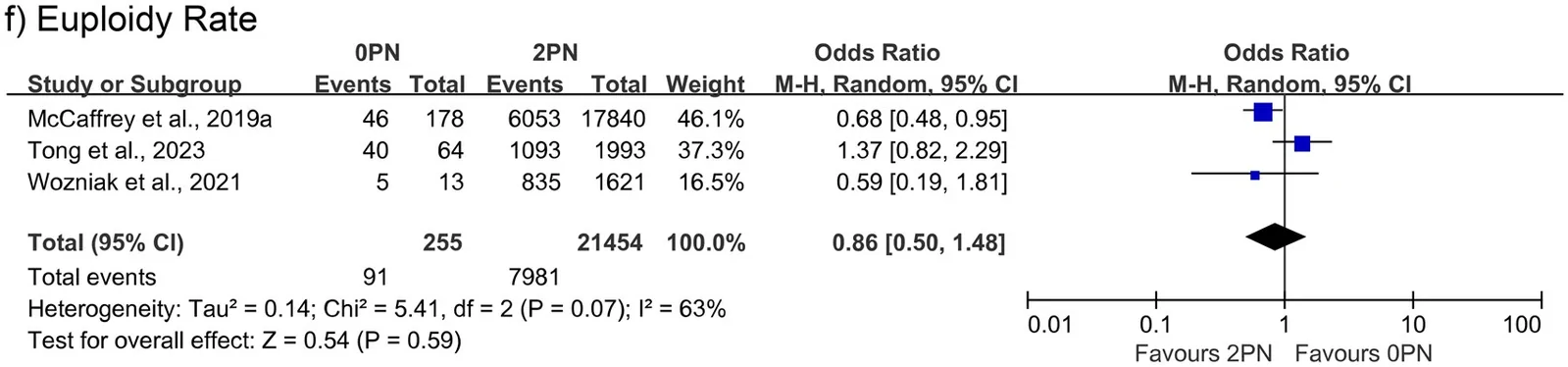
Continued.

##### IVF subgroup

There were only four studies with embryos inseminated by IVF (Hondo *et al.*, 2019; Fu *et al.*, 2021; Tong *et al.*, 2023; Zhu *et al.*, 2024b). There was significant difference between 0PN and 2PN embryos on the LBR in IVF with results favouring 2PN embryos (Fig. 3a; OR = 0.73, 95% CI = 0.58–0.93, *P*-value = 0.01; four studies, N = 7083; *I*^2^ = 0%).

**Figure 3.**
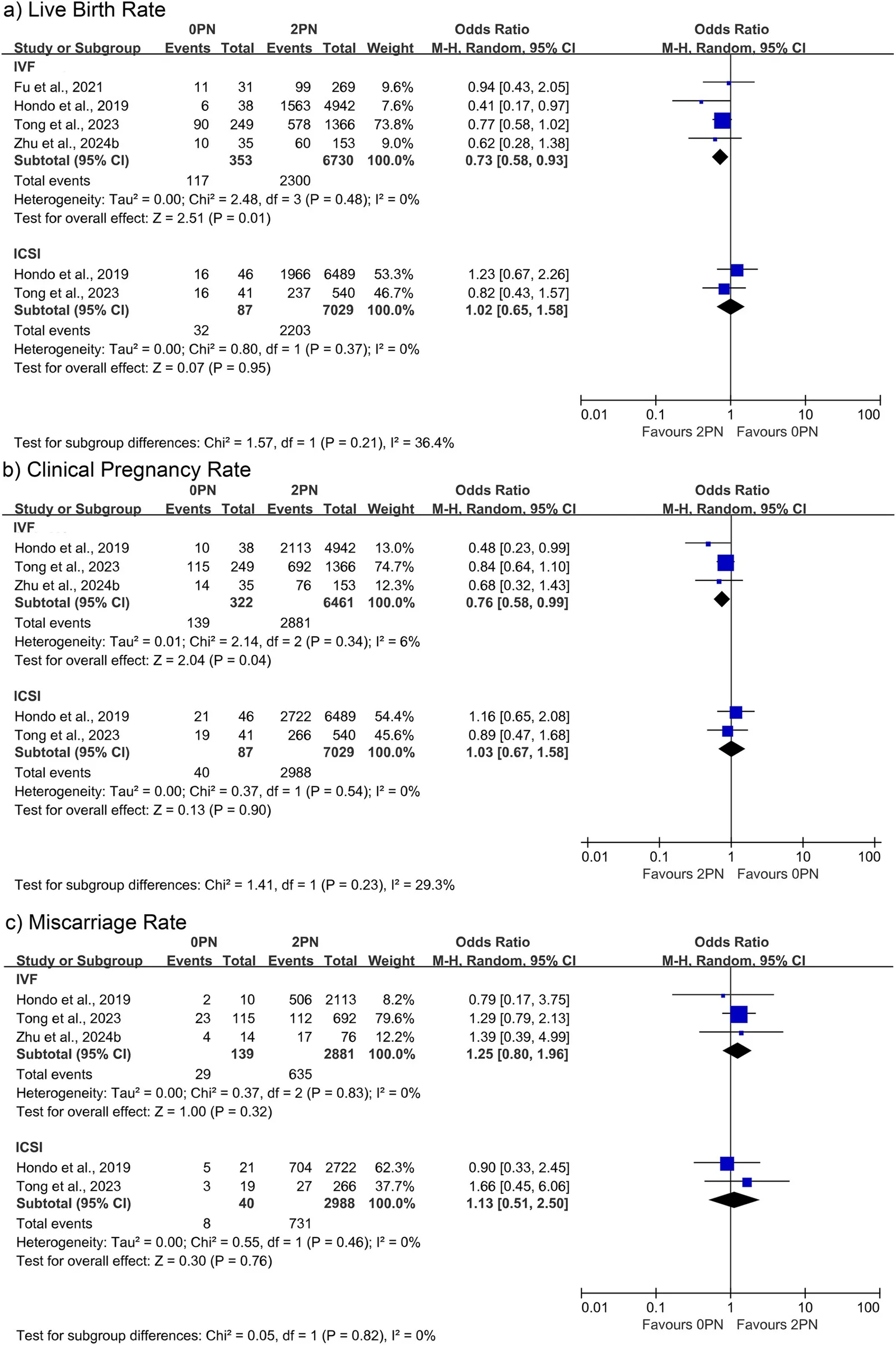
Subgroup analyses by insemination method (IVF versus ICSI) are presented where data permitted. Forest plots show the results of analyses comparing (a) live birth rate, (b) clinical pregnancy rate, (c) miscarriage rate, (d) malformation rate, and (e) blastulation rate between nonpronuclear (0PN) and dipronuclear (2PN) embryos. Each plot displays odds ratios (ORs) with 95% CIs for individual studies, with pooled estimates calculated using a random-effects model. Horizontal lines represent individual study estimates, and diamonds indicate overall effect sizes. Heterogeneity was assessed using the I2 statistic.

**Figure 3.**
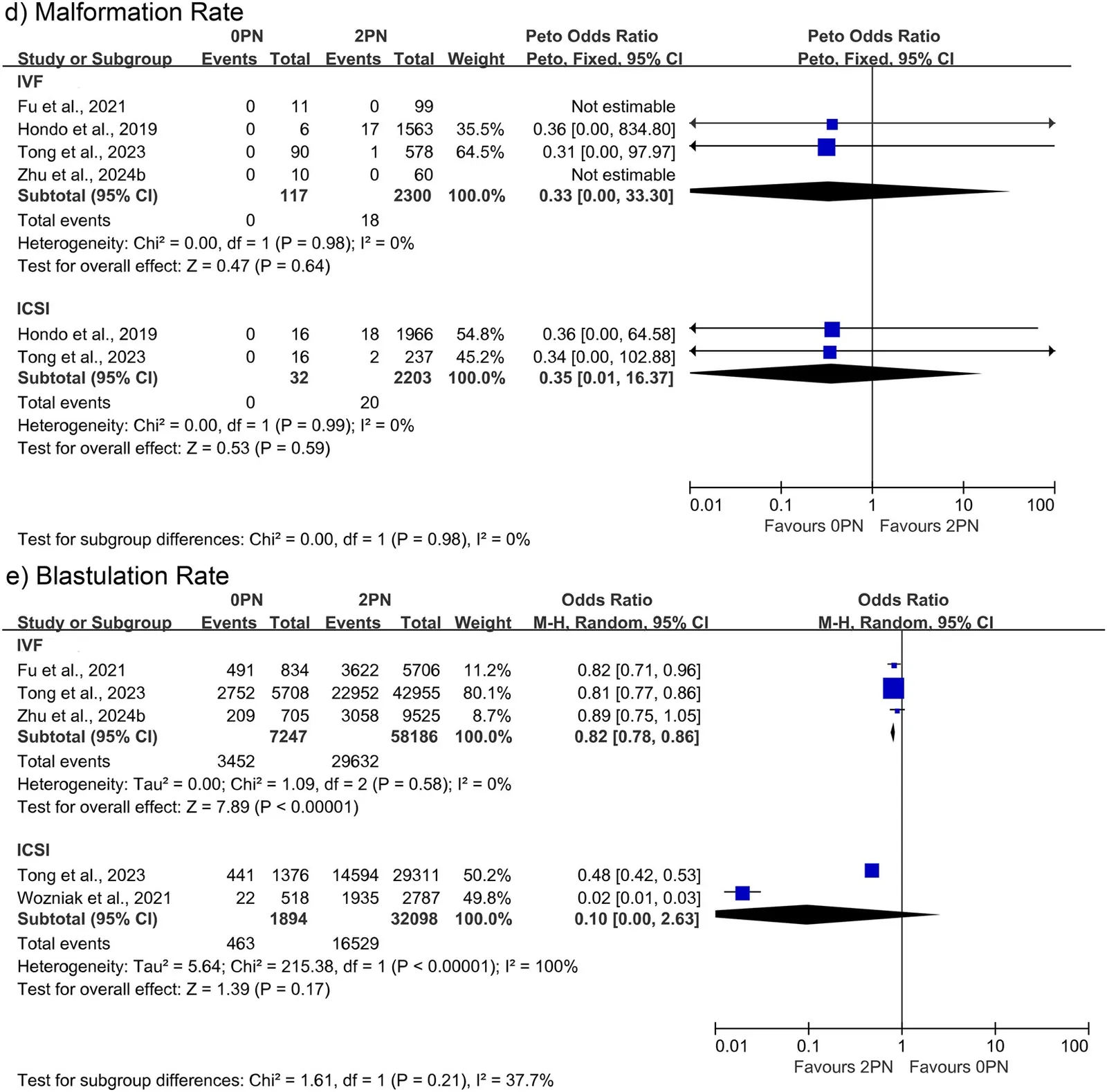
Continued.

##### ICSI subgroup

There were only two studies with embryos inseminated by ICSI (Hondo *et al.*, 2019; Tong *et al.*, 2023). There was no significant difference between 0PN and 2PN embryos on the LBR in ICSI (Fig. 3a; OR = 1.02, 95% CI = 0.65–1.58, *P*-value = 0.95; two studies, N = 7116; *I*^2^ = 0%).

#### Clinical pregnancy rate

Six of the 11 included studies reported CPRs after transferring 0PN embryos (Hondo *et al.*, 2019; Chen *et al.*, 2020; Li *et al.*, 2021; Tong *et al.*, 2023; Zhu *et al.*, 2024b; Xu *et al.*, 2025), and there was also no significant difference between 0PN and 2PN embryos on the overall CPR (Fig. 2b; OR = 0.96, 95% CI = 0.85–1.08, *P*-value = 0.48; six studies, N = 34 548; *I*^2^ = 0%). Both the asymmetry of the funnel plot (Supplementary Fig. S1b) and the Egger’s test (*Y* = −1.25, 95% CI = −3.13 to 0.63, *t* = 1.85, one-tailed *P*-value = 0.07) indicate possible publication bias.

##### IVF subgroup

Three included studies reported clinical pregnancies after transferring 0PN embryos inseminated by IVF (Hondo *et al.*, 2019; Tong *et al.*, 2023; Zhu *et al.*, 2024b). There was a significant difference between 0PN and 2PN embryos on the LBR in IVF, with results favouring 2PN embryos (Fig. 3b; OR = 0.76, 95% CI = 0.58–0.99, *P*-value =0.04; three studies, N = 6783; *I*^2^ = 6%).

##### ICSI subgroup

Two included studies reported clinical pregnancies after transferring 0PN embryos inseminated by ICSI (Hondo *et al.*, 2019; Tong *et al.*, 2023). There was no significant difference between 0PN and 2PN embryos on CPR (Fig. 3b; OR = 1.03, 95% CI = 0.67–1.58, *P*-value = 0.90; two studies, N = 7116; *I*^2^ = 0%).

#### Miscarriage rate

Six of the 11 included studies reported miscarriage rates after transferring 0PN embryos (Hondo *et al.*, 2019; Chen *et al.*, 2020; Li *et al.*, 2021; Tong *et al.*, 2023; Zhu *et al.*, 2024b; Xu *et al.*, 2025), and there was no significant difference between 0PN and 2PN embryos (Fig. 2c; OR = 1.04, 95% CI = 0.84–1.29, *P*-value = 0.70; six studies, N = 15 528; *I*^2^ = 0%). The asymmetry of the funnel plot indicates possible publication bias (Supplementary Fig. S1c), but the Egger’s test showed no evidence of publication bias (*Y* = 0.59, 95% CI = −1.35 to 2.53, *t* = 0.85, one-tailed *P*-value = 0.22).

##### IVF subgroup

Three included studies reported miscarriages after transferring 0PN embryos inseminated by IVF (Hondo *et al.*, 2019; Tong *et al.*, 2023; Zhu *et al.*, 2024b). There was no significant difference between 0PN and 2PN embryos on miscarriage rate (Fig. 3c; OR = 1.25, 95% CI = 0.80–1.96, *P*-value = 0.32; three studies, N = 3020; *I*^2^ = 0%).

##### ICSI subgroup

Two included studies reported miscarriages after transferring 0PN embryos inseminated by ICSI (Hondo *et al.*, 2019; Tong *et al.*, 2023). There was no significant difference between 0PN and 2PN embryos on miscarriage rate (Fig. 3c; OR = 1.13, 95% CI = 0.51–2.50, *P*-value = 0.76; two studies, N = 3028; *I*^2^ = 0%).

#### Malformation rate

Six of the 11 included studies reported malformation rates after transferring 0PN embryos (Hondo *et al.*, 2019; Fu *et al.*, 2021; Li *et al.*, 2021; Tong *et al.*, 2023; Zhu *et al.*, 2024b; Xu *et al.*, 2025), with two of the studies (Fu *et al.*, 2021; Zhu *et al.*, 2024b) having no event in both 0PN and 2PN group thus carrying no weight in the forest plot. There was no significant difference between 0PN and 2PN embryos on the overall malformation rate (Fig. 2d; OR = 1.05, 95% CI = 0.45–2.45, *P*-value = 0.91; six studies, N = 11 840; *I*^2^ = 0%). Both the asymmetry of the funnel plot (Supplementary Fig. S1d) and the Egger’s test (*Y* = −0.93, 95% CI = −1.46 to (−0.41)), *t* = 7.62, one-tailed *P*-value = 0.01) showed indications of publication bias.

##### IVF subgroup

Four included studies reported malformations after transferring 0PN embryos inseminated by IVF (Hondo *et al.*, 2019; Fu *et al.*, 2021; Tong *et al.*, 2023; Zhu *et al.*, 2024b), with two of the studies (Fu *et al.*, 2021; Zhu *et al.*, 2024b) having no event in both 0PN and 2PN groups, thus carrying no weight in the forest plot. There was no significant difference between 0PN and 2PN embryos on malformation rate in IVF (Fig. 3d; OR = 0.33, 95% CI = 0.00–33.3, *P*-value = 0.64; four studies, N = 2417; *I*^2^ = 0%).

##### ICSI subgroup

Two included studies reported malformations after transferring 0PN embryos inseminated by ICSI (Hondo *et al.*, 2019; Tong *et al.*, 2023). There was no significant difference between 0PN and 2PN embryos on malformation rate (Fig. 3d; OR = 0.35, 95% CI = 0.01–16.37, *P*-value = 0.59; two studies, N = 2235; *I*^2^ = 0%).

#### Blastulation rate

Four of the 11 included studies reported blastulation rates of 0PN embryos (Fu *et al.*, 2021; Wozniak *et al.*, 2021; Tong *et al.*, 2023; Zhu *et al.*, 2024b). There was a significant difference between 0PN and 2PN embryos, with results favouring 2PN embryos (Fig. 2e; OR = 0.34, 95% CI = 0.17–0.68, *P*-value = 0.002; four studies, N = 99 425; *I*^2^ = 99%). Asymmetry of the funnel plot indicates possible publication bias (Supplementary Fig. S1e), but the Egger’s test showed no evidence of publication bias (*Y* = −7.92, 95% CI = −41.34 to 25.50, *t* = 1.02, one-tailed *P*-value = 0.21).

##### IVF subgroup

Three included studies reported blastulation rate of 0PN embryos inseminated by IVF (Fu *et al.*, 2021; Tong *et al.*, 2023; Zhu *et al.*, 2024b). There was a significant difference between 0PN and 2PN embryos on blastulation rate in IVF with results favouring 2PN embryos (Fig. 3e; OR = 0.82, 95% CI = 0.78–0.86, *P*-value <0.001; three studies, N = 65 433; *I*^2^ = 0%).

##### ICSI subgroup

Two included studies reported blastulation rate of 0PN embryos inseminated by ICSI (Wozniak *et al.*, 2021; Tong *et al.*, 2023). There was no significant difference between 0PN and 2PN embryos on blastulation rate (Fig. 3e; OR = 0.10, 95% CI = 0.00–2.63, *P*-value = 0.17; two studies, N = 33 992; *I*^2^ = 100%).

#### Euploidy rate

Three of the 11 included studies reported euploidy rates of 0PN embryos (McCaffrey *et al.*, 2019a; Wozniak *et al.*, 2021; Tong *et al.*, 2023) and did not show any significant difference between 0PN and 2PN embryos (Fig. 2f; OR = 0.86, 95% CI = 0.50–1.48, *P*-value = 0.59; three studies, N = 21 709; *I*^2^ = 63%). Both the symmetry of the funnel plot (Supplementary Fig. S1f) and the Egger’s test (*Y* = 0.55, 95% CI = −41.39 to 42.49, *t* = 0.17, one-tailed *P*-value = 0.45) showed no evidence of publication bias.

##### IVF subgroup

See Supplementary Data File S3 for details.

##### ICSI subgroup

See Supplementary Data File S3 for details.

### Outcome comparison: 1PN embryos versus 2PN embryos

#### Live birth rate

Seven of the 11 included studies reported LBRs after transferring 1PN embryos (Hondo *et al.*, 2019; Chen *et al.*, 2020; Li *et al.*, 2021; Tong *et al.*, 2023; Labied *et al.*, 2024; Zhu *et al.*, 2024b; Xu *et al.*, 2025). The 1PN embryos have a significantly lower LBR compared to 2PN embryos (Fig. 4a; OR = 0.83, 95% CI = 0.70–0.98, *P*-value 0.03; seven studies, N = 34 584; *I*^2^ = 12%). Both the symmetry of the funnel plot (Supplementary Fig. S2a) and the Egger’s test (*Y* = −0.14, 95% CI = −2.46 to 2.18, *t* = 0.16, one-tailed *P*-value 0.44) showed no evidence of publication bias.

**Figure 4.**
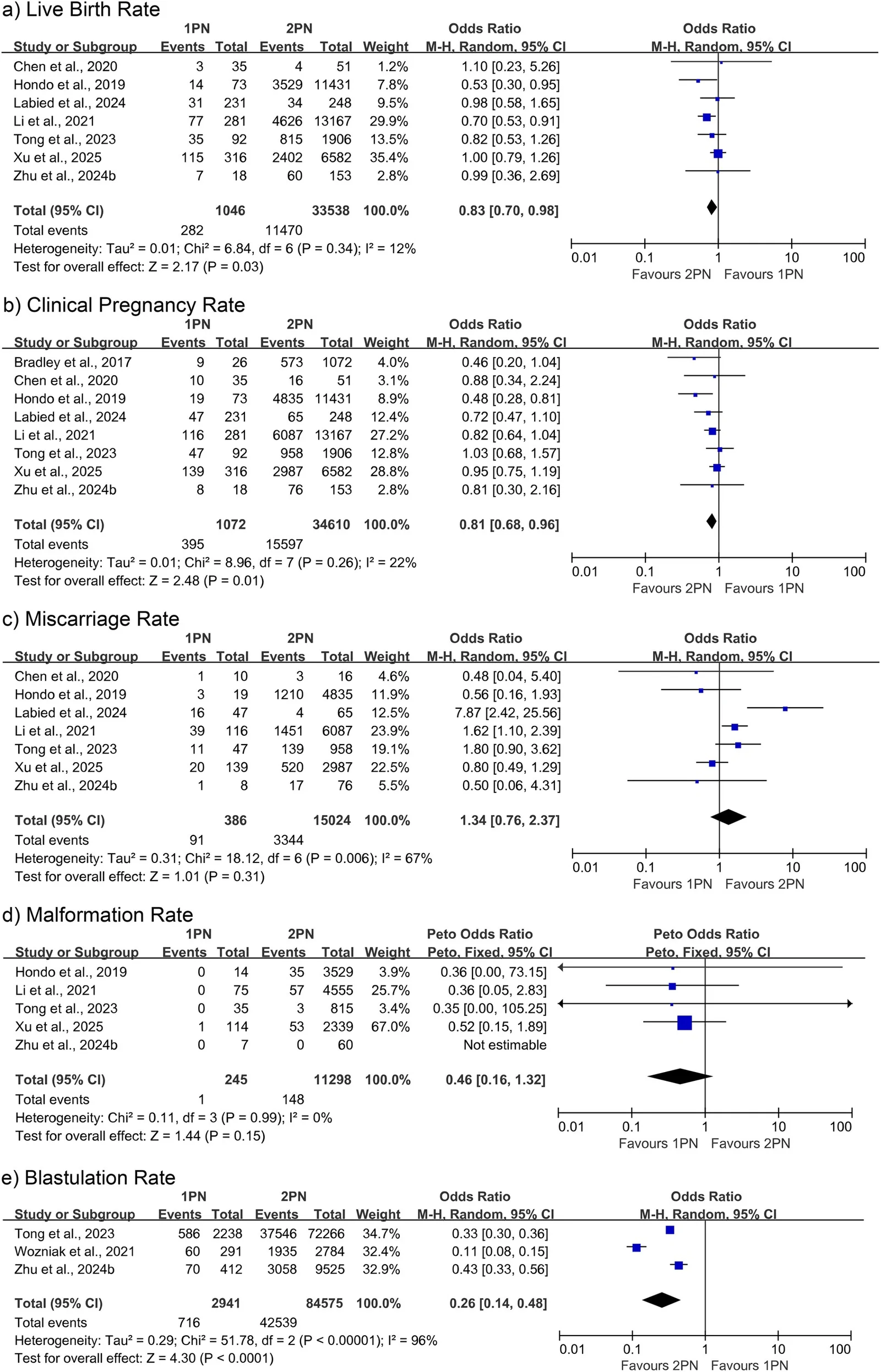
Comparison of outcomes between monopronuclear (1PN) embryos and dipronuclear (2PN) embryos. The 1PN embryos showed a significantly lower live birth rate, clinical pregnancy rate, and blastulation rate compared with 2PN embryos; however, no significant differences were observed in miscarriage rate, malformation rate, or euploidy rate (with or without confirmation of biparental inheritance for 1PN embryos) between 1PN and 2PN embryos. Forest plots of (a) live birth rate, (b) clinical pregnancy rate, (c) miscarriage rate, (d) malformation rate, (e) blastulation rate, (f) euploidy rate without bi-parental inheritance confirmation for 1PN embryo, and (g) euploidy rate with bi-parental inheritance confirmation for 1PN embryo between 1PN embryos and 2PN embryos. Each plot presents the odds ratios (ORs) with 95% CIs for individual studies, with pooled estimates calculated using a random-effects model. Horizontal lines represent individual study estimates, and diamonds represent overall effects. Heterogeneity was assessed using the I2 statistic.

**Figure 4.**
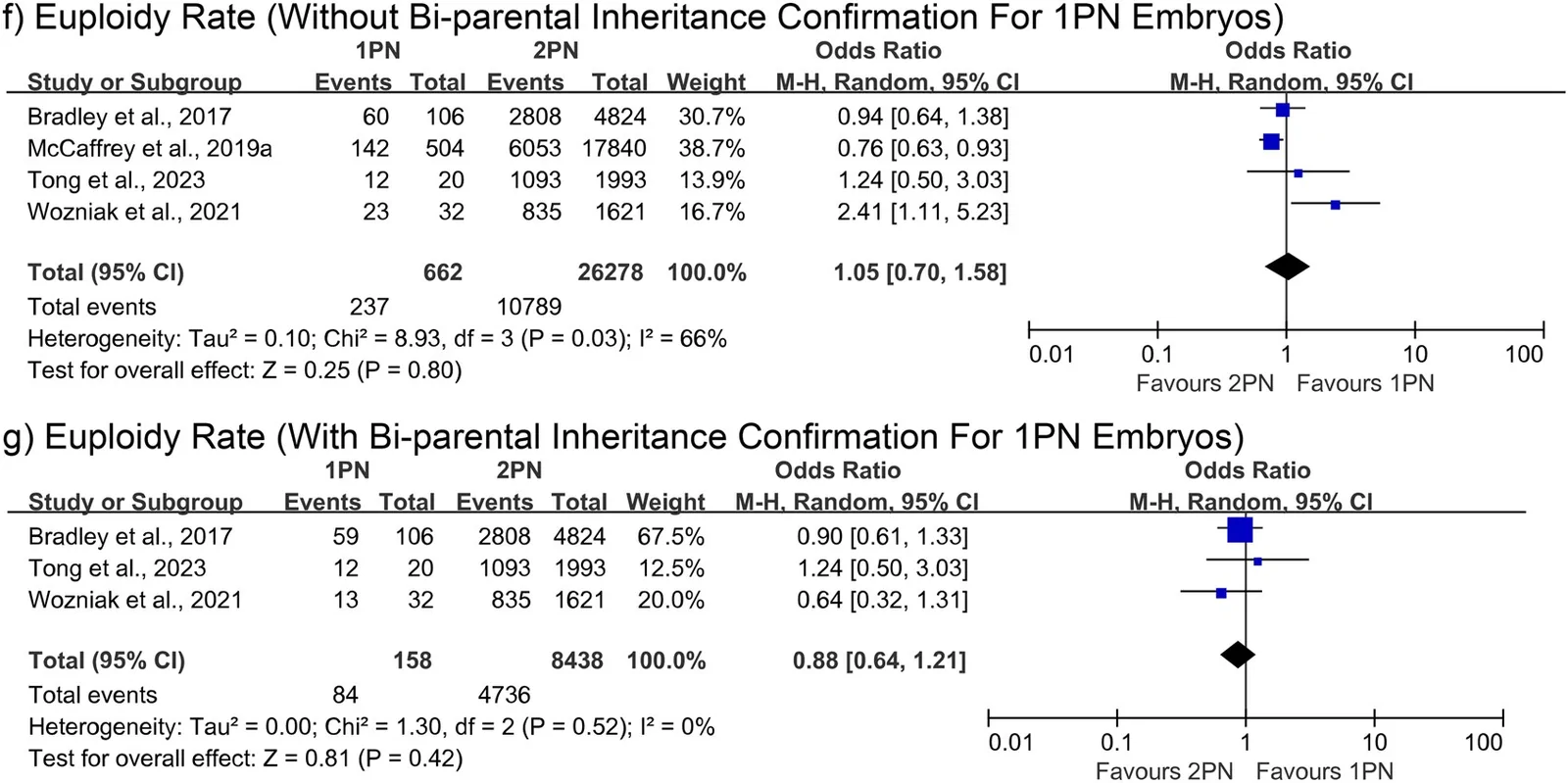
Continued.

##### IVF subgroup

Four included studies reported live births after transferring 1PN embryos inseminated by IVF (Hondo *et al.*, 2019; Tong *et al.*, 2023; Labied *et al.*, 2024; Zhu *et al.*, 2024b). There was no significant difference between 1PN and 2PN embryos on LBR (Fig. 5a; OR = 0.94, 95% CI = 0.68–1.28, *P*-value = 0.68; four studies, N = 6864; *I*^2^ = 0%).

**Figure 5.**
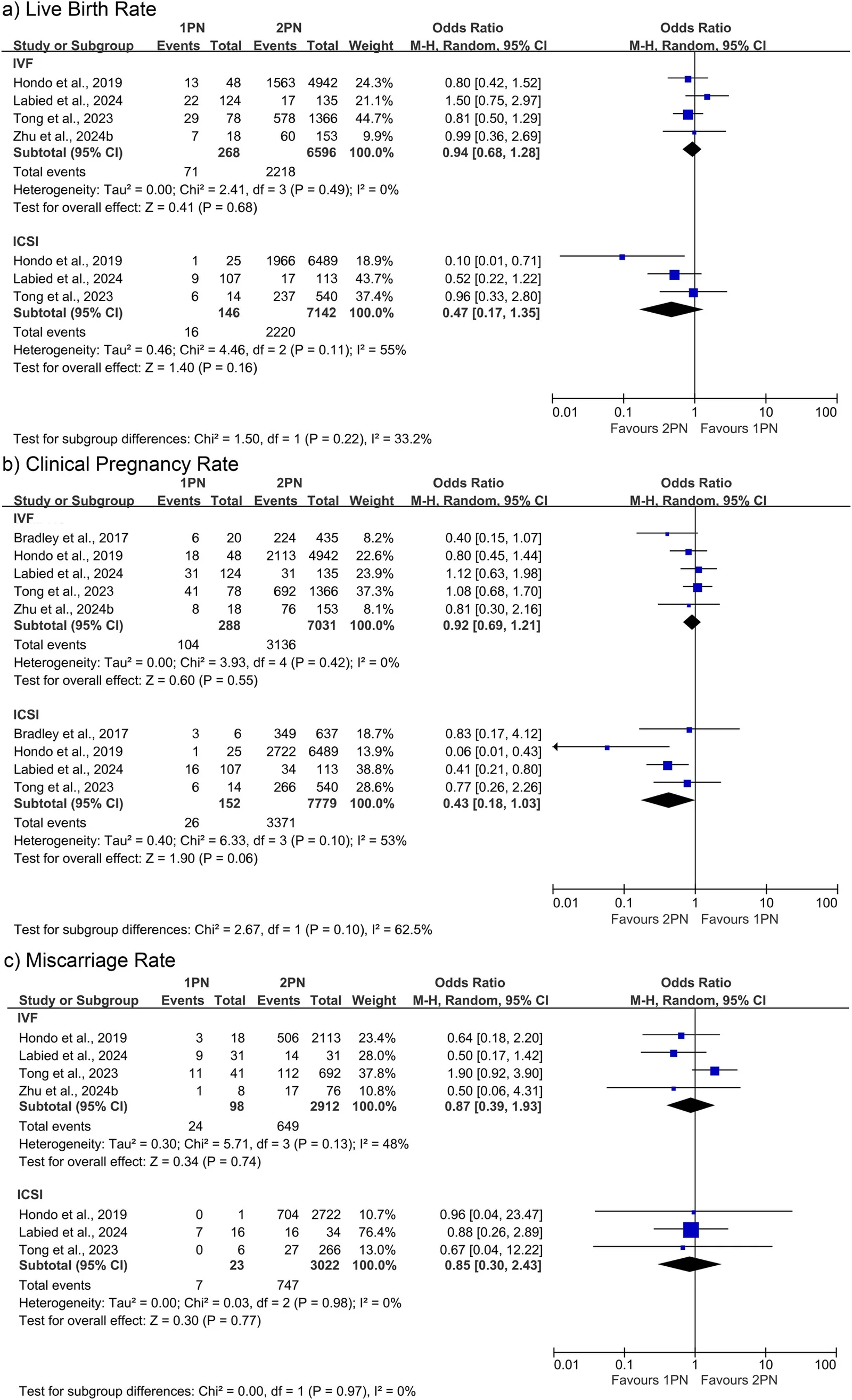
Subgroup analyses by insemination method (IVF versus ICSI) are presented where data permitted. Forest plots show the results of analyses comparing (a) live birth rate, (b) clinical pregnancy rate, (c) miscarriage rate, (d) malformation rate, and (e) blastulation rate for 1PN embryo between 1PN embryos and dipronuclear (2PN) embryos. Each plot displays odds ratios (ORs) with 95% CIs for individual studies, with pooled estimates calculated using a random-effects model. Horizontal lines represent individual study estimates, and diamonds indicate overall effect sizes. Heterogeneity was assessed using the I2 statistic.

**Figure 5.**
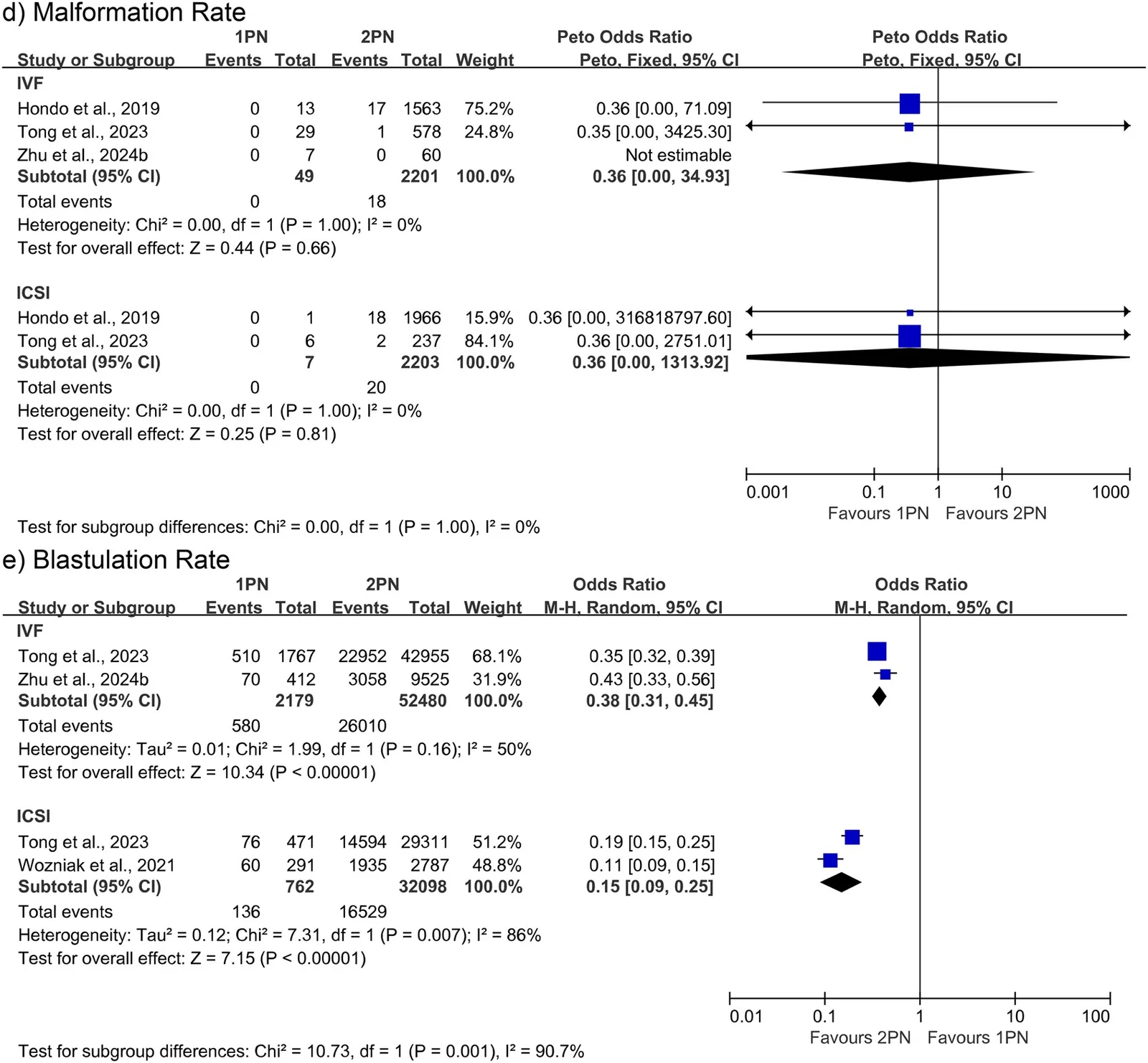
Continued.

##### ICSI subgroup

Three included studies reported live births after transferring 1PN embryos inseminated by ICSI (Hondo *et al.*, 2019; Tong *et al.*, 2023; Labied *et al.*, 2024) and showed no significant difference between 1PN and 2PN embryos on LBR (Fig. 5a; OR = 0.47, 95% CI = 0.17–1.35, *P*-value = 0.16; three studies, N = 7288; *I*^2^ = 55%).

#### Clinical pregnancy rate

Eight of the 11 included studies reported CPRs after transferring 1PN embryos (Bradley *et al.*, 2017; Hondo *et al.*, 2019; Chen *et al.*, 2020; Li *et al.*, 2021; Tong *et al.*, 2023; Labied *et al.*, 2024; Zhu *et al.*, 2024b; Xu *et al.*, 2025) and showed that 1PN embryos have a significantly lower CPR compared to 2PN embryos (Fig. 4b; OR = 0.81, 95% CI = 0.68–0.96, *P*-value 0.01; eight studies, N = 35 682; *I*^2^ = 22%). Both the symmetry of the funnel plot (Supplementary Fig. S2b) and the Egger’s test (*Y* = −1.09, 95% CI = −3.04 to 0.86, *t* = 1.36, one-tailed *P*-value 0.11) showed no evidence of publication bias.

##### IVF subgroup

Five included studies reported clinical pregnancies after transferring 1PN embryos inseminated by IVF (Bradley *et al.*, 2017; Hondo *et al.*, 2019; Tong *et al.*, 2023; Labied *et al.*, 2024; Zhu *et al.*, 2024b). There was no significant difference between 1PN and 2PN embryos on CPR (Fig. 5b; OR = 0.92, 95% CI = 0.69–1.21, *P*-value 0.55; five studies, N = 7319; *I*^2^ = 0%).

##### ICSI subgroup

Four included studies reported CPRs after transferring 1PN embryos inseminated by ICSI (Bradley *et al.*, 2017; Hondo *et al.*, 2019; Tong *et al.*, 2023; Labied *et al.*, 2024). There was no significant difference between 1PN and 2PN embryos on CPR (Fig. 5b; OR = 0.43, 95% CI = 0.18–1.03, *P*-value = 0.06; four studies, N = 7931; *I*^2^ = 53%).

#### Miscarriage rate

Seven of the 11 included studies reported miscarriage rates after transferring 1PN embryos (Hondo *et al.*, 2019; Chen *et al.*, 2020; Li *et al.*, 2021; Tong *et al.*, 2023; Labied *et al.*, 2024; Zhu *et al.*, 2024b; Xu *et al.*, 2025). There was no significant difference between 1PN and 2PN embryos (Fig. 4c; OR = 1.34, 95% CI = 0.76–2.37, *P*-value = 0.31; seven studies, N = 15 410; *I*^2^ = 67%). Asymmetry of the funnel plot indicates possible publication bias (Supplementary Fig. S2c), but the Egger’s test showed no evidence of publication bias (*Y* = −0.22, 95% CI = −3.70 to 3.27, *t* = 0.16, one-tailed *P*-value = 0.44).

##### IVF subgroup

Four included studies reported miscarriages after transferring 1PN embryos inseminated by IVF (Hondo *et al.*, 2019; Tong *et al.*, 2023; Labied *et al.*, 2024; Zhu *et al.*, 2024b). There was no significant difference between 1PN and 2PN embryos on miscarriage rate (Fig. 5c; OR = 0.87, 95% CI = 0.39–1.93, *P*-value = 0.74; four studies, N = 3010; *I*^2^ = 48%).

##### ICSI subgroup

Three included studies reported miscarriages after transferring 1PN embryos inseminated by ICSI (Hondo *et al.*, 2019; Tong *et al.*, 2023; Labied *et al.*, 2024). There was no significant difference between 1PN and 2PN embryos on miscarriage rate (Fig. 5c; OR = 0.85, 95% CI = 0.30–2.43, *P*-value = 0.77; three studies, N = 3045; *I*^2^ = 0%).

#### Malformation rate

Five of the 11 included studies reported malformation rates after transferring 1PN embryos (Hondo *et al.*, 2019; Li *et al.*, 2021; Tong *et al.*, 2023; Zhu *et al.*, 2024b; Xu *et al.*, 2025), with one of the studies (Zhu *et al.*, 2024b) having no event in both 1PN and 2PN groups, thus carrying no weight in the forest plot. There was no significant difference in the overall malformation rate (Fig. 4d; OR = 0.46, 95% CI = 0.16–1.32, *P*-value = 0.15; five studies, N = 11 543; *I*^2^ = 0%). Asymmetry of the funnel plot indicates possible publication bias (Supplementary Fig. S2d), but the Egger’s test showed no evidence of publication bias (*Y* = −0.22, 95% CI = −0.95 to 0.52, *t* = 1.26, one-tailed *P*-value = 0.17).

##### IVF subgroup

Three included studies reported malformations after transferring 1PN embryos inseminated by IVF (Hondo *et al.*, 2019; Tong *et al.*, 2023; Zhu *et al.*, 2024b), with one of the studies (Zhu *et al.*, 2024b) having no event in both 1PN and 2PN groups, thus carrying no weight in the forest plot. There was no significant difference between 1PN and 2PN embryos on malformation rate (Fig. 5d; OR = 0.36, 95% CI = 0.00–34.93, *P*-value = 0.66; three studies, N = 2250; *I*^2^ = 0%).

##### ICSI subgroup

Two included studies reported malformations after transferring 1PN embryos inseminated by ICSI (Hondo *et al.*, 2019; Tong *et al.*, 2023). There was no significant difference between 1PN and 2PN embryos on malformation rate (Fig. 5d; OR = 0.36, 95% CI = 0.00–1313.92, *P*-value = 0.81; two studies, N = 2210; *I*^2^ = 0%).

#### Blastulation rate

Four of the 11 included studies reported the blastulation rate of 1PN embryos (Wozniak *et al.*, 2021; Tong *et al.*, 2023; Zhu *et al.*, 2024b); however, Chen *et al.* (2020) was not used for analysis (see Supplementary Data File S2). There was a significant difference between 1PN and 2PN embryos’ results favouring 2PN embryos (Fig. 4e; OR = 0.26, 95% CI = 0.14–0.48, *P*-value <0.001; three studies, N = 87 516; *I*^2^ = 96%). Asymmetry of the funnel plot indicates possible publication bias (Supplementary Fig. S2e), but the Egger’s test showed no evidence of publication bias (*Y* = −4.12, 95% CI = −100.00 to 91.76, *t* = 0.54, one-tailed *P*-value = 0.34).

##### IVF subgroup

Two included studies reported blastulation rate of 1PN embryos inseminated by IVF (Tong *et al.*, 2023; Zhu *et al.*, 2024b). There was a significant difference between 1PN and 2PN embryos on blastulation rate in IVF with results favouring 2PN embryos (Fig. 5e; OR = 0.38, 95% CI = 0.31–0.45, *P*-value <0.001; two studies, N = 54 659; *I*^2^ = 50%).

##### ICSI subgroup

Two included studies reported a blastulation rate of 1PN embryos inseminated by ICSI (Wozniak *et al.*, 2021; Tong *et al.*, 2023). There was a significant difference between 1PN and 2PN embryos on blastulation rate in ICSI, with results favouring 2PN embryos (Fig. 5e; OR = 0.15, 95% CI = 0.09–0.25, *P*-value <0.001; two studies, N = 32 860; *I*^2^ = 86%).

#### Euploidy rate

Four of the 11 included studies reported a euploidy rate of 1PN embryos (Bradley *et al.*, 2017; McCaffrey *et al.*, 2019a; Wozniak *et al.*, 2021; Tong *et al.*, 2023), and there was no significant difference between 1PN and 2PN embryos (Fig. 4f; OR = 1.05, 95% CI = 0.70–1.58, *P*-value = 0.80; four studies, N = 26 940; *I*^2^ = 66%). Both the asymmetry of the funnel plot (Supplementary Fig. S2f) and the Egger’s test (*Y* = 2.48, 95% CI = −1.46 to 6.42, *t* = 2.70, one-tailed *P*-value = 0.06) showed indications of publication bias.

##### IVF subgroup

See Supplementary Data File S3 for details.

##### ICSI subgroup

See Supplementary Data File S3 for details.

#### Euploidy rate with bi-parental inheritance confirmation for 1PN embryos

Three of the 11 included studies reported bi-parental inheritance confirmation of 1PN embryos (Bradley *et al.*, 2017; Wozniak *et al.*, 2021; Tong *et al.*, 2023). The rate of 1PN embryos that were euploid and with confirmed bi-parental inheritance was not significantly different from the euploidy rate of 2PN embryos (Fig. 4g; OR = 0.88, 95% CI = 0.64–1.21, *P*-value = 0.42; three studies, N = 8596; *I*^2^ = 0%).

##### IVF subgroup

See Supplementary Data File S3 for details.

##### ICSI subgroup

See Supplementary Data File S3 for details.

### Sensitivity analyses

There were no differences in conclusions after performing sensitivity analyses using the number of embryos transferred as the denominator in place of the original denominators (see Supplementary Fig. S3). There remained no significant differences in miscarriage rate (OR = 1.01, 95% CI = 0.83–1.23, *P*-value = 0.92; six studies, N = 34 548; *I*^2^ = 0%) and malformation rate (OR = 0.80, 95% CI = 0.23–2.82, *P*-value = 0.73; five studies, N = 27 801; *I*^2^ = 0%) between 0PN and 2PN embryos, and no significant differences in miscarriage rate (OR = 1.12, 95% CI = 0.69–1.84, *P*-value = 0.64; seven studies, N = 34 584; *I*^2^ = 62%) and malformation rate (OR = 0.46, 95% CI = 0.17–1.24, *P*-value = 0.12; five studies, N = 34 019; *I*^2^ = 0%) between 1PN and 2PN embryos.

### Overall certainty of evidence

The certainty of evidence for LBR, CPR, miscarriage rate, malformation rate, blastulation rate, and euploidy rate of 0PN embryos compared to 2PN embryos, as well as 1PN embryos compared to 2PN embryos, are summarized in Table 2. The summary of findings table included 0PN embryos versus dipronuclear (2PN) embryos and 1PN embryos versus dipronuclear (2PN) embryos.

#### Outcome comparison: 0PN embryos versus 2PN embryos

The certainty of evidence for LBR and CPR was graded as moderate due to potential risk of bias. The certainty for miscarriage rate was rated as low due to both the risk of bias and imprecision arising from the limited number of reported events. For malformation rate, blastulation rate, and euploidy rate, the certainty of evidence was deemed very low. Reasons for downgrading included potential risk of bias, substantial heterogeneity, imprecision related to the small number of events, and the presence of wide CIs.

#### Outcome comparison: 1PN embryos versus 2PN embryos

The certainty of evidence for CPR was moderate due to the potential risk of bias. The certainty of evidence for LBR was low, downgraded due to the potential risk of bias and imprecision related to the small number of events. The certainty of evidence for miscarriage rate, malformation rate, euploidy rate, and blastulation rate was very low. Reasons for downgrading included potential risk of bias, substantial heterogeneity, imprecision related to the small number of events, and the presence of wide CIs.

## Discussion

The observation of 2PN and 2PB has long been the standard for determining normal fertilization in human ARTs. The discovery that embryos derived from zygotes with atypical PN numerations can have a normal diploid genome, despite at a lower percentage compared to their 2PN counterparts (Staessen and Van Steirteghem, 1997; Yan *et al.*, 2005; Campos *et al.*, 2007), has sparked a prolonged debate on whether PN numeration should be the sole indication of normal fertilization and whether 0PN and 1PN embryos are viable candidates for clinical use.

Findings on the effect of transferring 0PN and 1PN embryos are limited due to the low occurrence of viable embryos derived from 0PN and 1PN zygotes, and even fewer have been transferred. Early case reports provided limited data and often lacked follow-up on neonatal outcomes (Gras and Trounson, 1999; Burney *et al.*, 2008). More studies with larger sample sizes have emerged in recent years demonstrating that 0PN and 1PN embryos, despite having lower developmental potential, those that reach the blastocyst stage can have similar clinical outcomes compared to their 2PN counterparts (Tong *et al.*, 2023; Zhu *et al.*, 2024a,b; Xu *et al.*, 2025). Therefore, this study aims to study the effect of transferring 0PN and 1PN embryos on clinical outcomes and euploidy rates and shed light on whether these embryos are clinically worthwhile for transfer.

A total of 11 publications met the inclusion criteria for this review. Two studies reported embryo euploidy outcomes only, seven studies reported embryo transfer outcomes only, and two studies reported both embryo euploidy and embryo transfer outcomes.

### Transfer of 0PN embryos

Our study found that transferring 0PN embryos has no significant difference in the overall LBR, clinical pregnancy, miscarriage rate, malformation rate, and euploidy rate. However, in the IVF subgroup, transferring of 0PN has a significantly lower LBR and CPR. The heterogeneity between studies on the clinical outcomes between 0PN and 2PN embryos is relatively low, except for euploidy rates, which have moderate heterogeneity between studies. This is likely because a sperm is injected into the oocyte when inseminated by ICSI, reducing the ambiguity around fertilization origin and decreasing the risk of polyspermy, which is more difficult to exclude in IVF-derived 0PN zygotes.

### Transfer of 1PN embryos

Transferring 1PN embryos resulted in a significantly lower LBR and clinical pregnancy, while there was no significant effect seen on the miscarriage rate and malformation rate and euploidy rate. The heterogeneity between studies on the clinical outcomes between 1PN and 2PN embryos is relatively low except for the miscarriage rate and euploidy rate, which have moderate heterogeneity.

However, it is not sufficient to only look at the euploidy rate of 1PN embryos. Since euploid 1PN embryos can arise from the parthenogenetic activation of oocytes and only carry genetic materials from one parent (Sultan *et al.*, 1995). There has been a report of a complete molar pregnancy after transfer of a euploid 1PN embryo (Zhou *et al.*, 2021). Complete hydatidiform moles have a genetic constitution that is solely paternally derived (Fan *et al.*, 2002). In most cases, it is the result of fertilization of an empty oocyte, which does not carry any genetic materials by a haploid sperm, then the haploid genome duplicates resulting in a diploid genome (Kalogiannidis *et al.*, 2018). Parthenogenetic embryos that carry two sets of maternal genomes are generally not viable due to the instability of maternally imprinted genes (Mai *et al.*, 2007).

Of the four studies that reported the euploidy rate of 1PN embryos, three studies investigated the bi-parental inheritance of these embryos (Bradley *et al.*, 2017; Wozniak *et al.*, 2021; Tong *et al.*, 2023). The rate of euploid 1PN embryos with bi-parental inheritance was not significantly different than the euploidy rate of 2PN embryos. There was no significant difference in both IVF and ICSI subgroup as well.

Since transfers of 1PN embryos have a significantly lower LBR and CPRs, and there is a potential risk of developing into complete molar pregnancies, additional information may be needed to determine whether a 1PN embryo is suitable for transfer. For example, euploid blastocysts have been reported to have a significantly greater expansion rate compared to aneuploid blastocysts (Huang *et al.*, 2019), and 1PN zygotes with PN area or diameter ≥710 μm^2^ or 31 μm, respectively, have similar developmental potential as 2PN zygotes (Araki *et al.*, 2018). Also, it has been reported that 1PN embryos derived from ICSI that reached the blastocyst stage have similar morphokinetics when compared to 2PN embryos (Mateo *et al.*, 2020). However, there is currently no substantial evidence showing that viable 1PN embryos can be differentiated through morphology or morphokinetics alone. Therefore, PGT-A with biparental inheritance confirmation for 1PN embryos prior to transfer is advisable when such testing is available and feasible.

In addition, embryos derived from APZs have a significantly lower blastulation rate than 2PN embryos. Therefore, extended culture to the blastocyst stage should be carried out for 0PN and 1PN embryos when considering the transfer of these embryos, as they are more likely to arrest at the cleavage stage and do not form viable embryos compared to normally fertilized embryos.

More research in the future is needed to compare the clinical and neonatal outcomes of confirmed euploid embryos derived from APZs versus euploid 2PN embryos. Such studies would provide more direct insight into whether pronuclear configuration itself has additional risks beyond chromosomal status. At present, such available literature is limited and lacks systematic neonatal and long-term follow-up data. Well-designed studies with PGT-A as well as confirmation of biparental inheritance and comprehensive clinical and neonatal outcome reporting are needed to determine whether embryos derived from abnormal pronuclear zygotes that are chromosomally normal carry any additional risk compared to euploid 2PN embryos.

### Limitations

There were high variations in the time at which fertilization assessment takes place among studies, particularly in studies where time-lapse monitoring of embryos was used, and late fertilization checking was often used to ensure there is no late appearance of PN for 0PN and 1PN embryos (Kobayashi *et al.*, 2021). However, to ensure a fair comparison can be conducted, the time of fertilization checking has been limited to 16–18 h post insemination as suggested in Istanbul consensus workshop on embryo assessment (Balaban *et al.*, 2011) and ESHRE guidelines for good practice in IVF laboratories (2016) for our study. Therefore, the results from this meta-analysis might only apply to laboratories using conventional incubators and embryo fertilization assessment under an inverted microscope during 16–18 h post-insemination.

Single time-point observations remain a major limitation in many studies included in this meta-analysis, as pronuclei that appeared and disappeared outside this narrow observation window may have been missed, leading to potential misclassification of fertilization status. With the increasing use of time-lapse monitoring systems that allow continuous monitoring of embryos, fertilization assessment can be performed more accurately. Kobayashi *et al.* (2021) demonstrated that combining time-lapse monitoring with short insemination protocols in conventional IVF cycles resulted in no 0PN-derived embryos being observed, suggesting that 0PN embryos reported in conventional IVF protocols are most likely due to missed PN observation rather than true 0PN fertilization.

Beyond limitations in fertilization assessment, the overall certainty of the evidence for live birth and CPRs ranged from moderate to low, while that for the remaining outcomes was very low (Table 2). Reasons for the downgraded certainty of the evidence were potential risk of bias, substantial heterogeneity, small number of events, and wide CI. The studies included were all observational, as randomized controlled trials would not be feasible given the low occurrence of APZs and the fact that different centres follow their own policies regarding the transfer of APZ-derived embryos. Of the nine included studies that reported outcomes after transfers of APZ-derived embryos, only one study (Labied *et al.*, 2024) specified that embryos selected for transfer were based solely on morphology and development, regardless of the number of PN observed. Two studies (Li *et al.*, 2021; Xu *et al.*, 2025) excluded 1PN embryos derived from ICSI; one centre (Bradley *et al.*, 2017) only transferred 1PN embryos after PGT-A with confirmation of biparental inheritance; and two studies (Hondo *et al.*, 2019; Zhu *et al.*, 2024b) explicitly stated that 2PN embryos were prioritized for transfer. These selection practices make it difficult to determine whether observed outcomes reflect true biological differences or the consequences of selective transfer. Such decision-making introduces unavoidable bias due to confounding by indication, which cannot be fully addressed through statistical adjustment. Also, most studies did not report and adjust for confounding factors, such that the risk of confounding bias was serious in most studies. Only two studies (Li *et al.*, 2021; Xu *et al.*, 2025) reported adjusted ORs on LBR, CPR, and miscarriage rate (see Supplementary Tables S2 and S3), and there were no differences in significance between unadjusted and adjusted ORs. Given the well-established effect of advanced maternal age on oocyte aneuploidy (Munné *et al.*, 1995; Hassold and Hunt, 2001; Nagaoka *et al.*, 2012; Verdyck *et al.*, 2023), the absence of maternal age adjustment in studies reporting euploidy rates represents an important limitation. Therefore, the reported euploidy outcomes should be interpreted with caution. The sample sizes of some studies are small, resulting in wide CIs for some outcomes. Heterogeneity was not substantial for most outcomes, but the *I*^2^ statistic has a substantial bias when there are less than seven studies included for analysis (von Hippel, 2015). In addition, the power of using funnel plot asymmetry to test for publication biases when there are fewer than 10 studies included in the analysis of outcomes is low (Sterne and Egger, 2001).

Finally, although a limited set of search terms was used for the literature search, the use of alternative terminology resulted in fewer relevant records for inclusion and did not identify any additional eligible studies.

### Agreements and disagreements with other studies or reviews

To date, only one systematic review and meta-analysis has evaluated clinical outcomes following the transfer of 0PN and 1PN blastocysts (Wu *et al.*, 2025). The review reported that the transfer of 1PN embryos was associated with significantly lower live birth and CPRs compared to 2PN embryos, and this effect remained significant within the ICSI subgroup. In contrast, we observed no significant differences in LBR or CPR within the ICSI subgroup. Similarly, Wu *et al.* found that the transfer of 0PN embryos resulted in a significantly lower LBR and CPR compared to 2PN embryos. While our overall analysis did not reveal significant differences between 0PN and 2PN embryo transfers, our subgroup analysis showed similar findings as Wu *et al.* (2025), where 0PN embryos have a significantly lower LBR and CPR in the IVF subgroup but not in the ICSI subgroup.

The discrepancies in findings between our study and that of Wu *et al.* (2025) may be attributed to differences in inclusion criteria. Specifically, our review excluded studies that involved double embryo transfers when data on SETs were not separately reported, as well as studies in which fertilization assessments were conducted outside the 16–18 h post-insemination window. In contrast, these studies were included in the analysis by Wu *et al.* (2025), which may have contributed to the divergent outcomes.

## Conclusion

Viable 0PN embryos are frequently attributed to missed pronuclear (PN) visualization due to the limitations of single time-point fertilization assessments. Our analysis demonstrates that 0PN-derived blastocysts do not differ significantly from 2PN counterparts in terms of clinical outcomes or euploidy rates. However, when derived from conventional IVF, 0PN embryos are associated with significantly lower live birth and CPRs, potentially due to undetected polyspermy.

For 1PN embryos, a significantly lower LBR and CPR were observed overall, though no significant differences were noted in miscarriage rate, malformation rate, or euploidy rate. While these findings suggest that 0PN and 1PN embryos may represent a viable option in specific clinical circumstances, they must be interpreted with extreme caution. The available literature is subject to strong and unresolvable confounding by indication. Furthermore, there remains a small but important risk that 1PN embryos may arise from parthenogenetic activation, which can result in a complete molar pregnancy. Consequently, the decision to transfer 1PN embryos should be individualized, taking into account patient-specific circumstances, the availability of alternative embryos, and access to genetic testing. When 1PN embryos are considered for transfer, patients should be counselled regarding the reduced implantation potential, the risk of molar pregnancy, and the potential benefits of PGT-A with confirmation of biparental inheritance when accessible.

## Supplementary Material

dmag015_Supplementary_Data

## Data Availability

The data underlying this review are available in the article and online supplementary data files.
