# Supplementary material for: Clinical outcomes and euploidy rates of embryos derived from zygotes with nonpronuclear (0PN) and monopronuclear (1PN) in IVF or ICSI cycles: a systematic review and meta-analysis
Source: Hum Reprod Update. 2026 Jun 4;32(6):693–716. doi: 10.1093/humupd/dmag015 (PMC13731786; doi:10.1093/humupd/dmag015)
Supplement: Supplementary file 1 [file dmag015_supplementary_data.docx]

Title: **Clinical outcomes and euploidy rates of embryos derived from zygotes with nonpronuclear (0PN) and monopronuclear (1PN) for in-vitro fertilisation (IVF) and intracytoplasmic sperm injection (ICSI) cycles: a systematic review and meta-analysis**

Running title: 0PN and 1PN for IVF and ICSI

Full author list: Hoi Wing CHAN^1^, Chi Chiu WANG^2*^, Yiu Leung David CHAN^1^

^1^Assisted Reproductive Technology Unit, Department of Obstetrics and Gynaecology, Faculty of Medicine, The Chinese University of Hong Kong

^2^Department of Obstetrics and Gynaecology, The Chinese University of Hong Kong, Hong Kong, China

^3^Author ORCID iD: 0009-0007-0564-3143; e-mail: hoiwingchan@cuhk.edu.hk

^4^Author ORCID iD: 0000-0002-3928-7278; e-mail: ccwang@cuhk.edu.hk

^5^Author ORCID iD:; 0000-0002-4736-8262; e-mail: [drdcyl16@cuhk.edu.hk](mailto:drdcyl16@cuhk.edu.hk)

*Correspondence address: Department of Obstetrics & Gynaecology, 1/F, Old Block (E Wing), Prince of Wales Hospital, Shatin, NT, Hong Kong SAR; e-mail: [ccwang@cuhk.edu.hk](mailto:ccwang@cuhk.edu.hk); ORCID iD: 0000-0002-3928-7278

**Table of contents**

Supplementary Data File S1. Databases search strategy

Supplementary Data File S2. Characteristics of included studies

Supplementary Table S1. Characteristics of excluded studies

Supplementary Figure S1. Funnel plots comparing outcomes between 0PN and 2PN embryos

Supplementary Figure S2. Funnel plots comparing outcomes between 1PN and 2PN embryos

Supplementary Data File S3. Subgroup analyses for euploidy rate

Supplementary Figure S3. Sensitivity analyses

Supplementary Table S2. Summary of adjusted odds ratios from Li et al. (2021)

Supplementary Table S3. Summary of adjusted odds ratios from Xu et al. (2025)

References

Supplementary Data File S1. Databases search strategy

**Cochrane Central Register of Controlled Studies (CENTRAL) search strategy**

Searched on 10 November 2025

#1 0PN (3)

#2 1PN (8)

#3 #1 OR #2 (9)

### **Embase search strategy**

OVID platform

Searched from 1910 to 10 November 2025

1 0PN.mp. (117)

2 1PN.mp. (313)

3 1 or 2 (347)

### **MEDLINE(R) ALL search strategy**

OVID platform

Searched from 1946 to 10 November 2025

1 0PN.mp. (83)

2 1PN.mp. (214)

3 1 or 2 (250)

### **PubMed search strategy**

Searched on 10 November 2025

(0PN) OR (1PN) (293)

**Fertility and Sterility search strategy**

Searched on 10 November 2025

[All: 0pn] (44)

[All: 1pn] (148)

Supplementary Data File S2. Characteristics of included studies [ordered by study ID]

Bradley et al., 2017

|  | ***Study characteristics*** | | |  |
| --- | --- | --- | --- | --- |
|  | Methods | - Retrospective study - Multi‐centre study performed at the Genea fertility clinics located at Sydney City, Liverpool, North West and Canberra - Oocyte retrieval cycles between 1^st^ June 2013 and 31^st^ August 2016 - Frozen-thawed embryo transfer between 10^th^ July 2013 and 13^th^ October 2016 | |  |
|  | Participants | - Median age (IQR):   PGT-1PN-IVF 37.5 (34.3–40.2), PGT-1PN-ICSI 36.2 (33.7–40.1),  PGT-2PN-IVF 36.5 (34.0–39.1), PGT-2PN-ICSI 36.7 (33.7–39.4),  FET-1PN-IVF 38.2 (34.2–40.3), FET -1PN-ICSI 37.0 (35.5–40.5),  FET-2PN-IVF 36.6 (33.9–38.9), FET-2PN-ICSI 36.5 (33.5–39.1)   - Inclusion criteria: 1PN or 2PN embryos from IVF or ICSI cycles; only patients who underwent PGT were included since 1PN embryos were routinely discarded - Exclusion criteria: donor-recipient cases; single gene disorder and chromosome translocation cases | |  |
|  | Interventions | - 1PN or 2PN embryos from IVF or ICSI insemination - Trophectoderm biopsy was performed on day 5 or 6 - Chromosomal analysis was performed by either CGH or NGS - Biparental inheritance was confirmed by identifying both maternal and paternal allele across chromosomes in the embryo genomes - Frozen-thawed single blastocyst transfer | |  |
|  | Outcomes | Secondary outcomes:   - Clinical pregnancy rate: defined as the detection of fetal heart motion by ultrasound about 4 weeks after embryo transfer per blastocyst transfers - Normal 1PN embryos: CGH or NGS reveals no detectable abnormalities, and bi-parental inheritance is confirmed - Blastulation rate has been adjusted for maternal age | |  |
|  | Notes |  | |  |
|  | ***Risk of bias*** |  | |  |
| Bias | | ROBINS‐E judgement | Support for judgement | |
| Bias due to confounding | | High risk | Embryo transfer outcome   - Stratified analysis was performed on participants' fertilisation method - The following confounders were measured but not adjusted for: participants' age and embryo quality, due to few 1PN embryo transfers and PGT reduces the influence of maternal age - The following confounders were not measured: participants' BMI, cause of infertility, the number of previous attempts, and cumulative dose of gonadotropin   Embryo ploidy outcome   - The following confounders were measured and adjusted for: participants' age - Stratified analysis was performed on participants' fertilisation method - The following confounders were measured but not adjusted for: participants' embryo quality - The following confounders were not measured: participants' body mass index (BMI), cause of infertility, the number of previous attempts, and cumulative dose of gonadotropin | |
| Bias arising from measurement of the exposure | | Some concerns | Measurement of exposure was well defined, but there could be error in single time-point assessment | |
| Bias in selection of participants into the study | | Low risk | Participants were not selected based on characteristics observed after the intervention began | |
| Bias due to post-exposure interventions | | Some concerns | - 1PN embryos were cultured individually - 2PN embryos were co-cultured | |
| Bias due to missing data | | Low risk | Outcomes of 1PN embryo transfer were obtained by telephone conversation with the mother/ through information provided by the doctor | |
| Bias in measurement of the outcomes | | Low risk | Blinding of outcome assessors was not reported but outcome measuring method was well defined and standardized. | |
| Bias in selection of the reported result | | Low risk | All measured outcomes were reported | |
| Overall bias | | High risk |  | |

Chen et al., 2020

|  | ***Study characteristics*** | | |  |
| --- | --- | --- | --- | --- |
|  | Methods | - Retrospective study - Consecutive sampling - Single‐centre study at the Jinhua People’s Hospital of China - From January 2014 to May 2018 | |  |
|  | Participants | - Mean age (SD): 33.4 (4.2) - Exclusion criteria: uterine malformations, uterine fibroids, or adenomyosis; history of recurrent pregnancy loss; history of hereditary or family diseases | |  |
|  | Interventions | - 0PN, 1PN or 2PN embryos from IVF or ICSI insemination - Fresh blastocyst transfer on day 5 or day 6 | |  |
|  | Outcomes | Primary outcomes   - Live birth rate: defined as number of deliveries with at least one live born baby per ART cycle   Secondary outcomes   - Clinical pregnancy: defined as the presence of a gestational sac with a fetal heartbeat on ultrasound examination 28 days after embryo transfer - Miscarriage: defined as loss of pregnancy before the 20th week of gestation - Blastulation rate: defined as the number of blastocysts formed per every fertilized zygote | |  |
|  | Notes | - Not all transfers are single embryo transfer, data from double embryo transfers were not included in the meta-analysis. - Data on blastulation rate were not used for analysis as only poor blastocysts were used as comparators. | |  |
|  | ***Risk of bias*** | | |  |
| Bias | | ROBINS‐E judgement | Support for judgement | |
| Bias due to confounding | | Very high  risk | Embryo transfer outcome   - The following confounders were measured but not adjusted for: participants' age, fertilisation method, and embryo quality - The following confounders were not measured: participants' BMI, cause of infertility, the number of previous attempts, and cumulative dose of gonadotropin | |
| Bias arising from measurement of the exposure | | Some concerns | Measurement of exposure was well defined, but there could be error in single time-point assessment | |
| Bias in selection of participants into the study | | Low risk | Participants were not selected based on characteristics observed after the intervention began; consecutive sampling was used in this study | |
| Bias due to post-exposure interventions | | Low risk | All embryos were cultured under the same conditions | |
| Bias due to missing data | | Low risk | All pregnancies were followed up until birth | |
| Bias in measurement of the outcomes | | Low risk | Blinding of outcome assessors was not reported but outcome measuring method was well defined and standardized. | |
| Bias in selection of the reported result | | Low risk | All measured outcomes were reported | |
| Overall bias | | High risk |  | |

Fu et al., 2021

|  | ***Study characteristics*** | | |  |
| --- | --- | --- | --- | --- |
|  | Methods | - Retrospective study - Single‐centre study at the Beijing Chao-Yang Hospital - From January 2018 to December 2019 | |  |
|  | Participants | - Mean age (SD): 0PN 32.2 (4.1), 2PN 32.5 (4.0) - Inclusion criterion: cycles with ≥4 oocytes - Exclusion criterion: cycles without cleaved 2PN or 0PN zygotes | |  |
|  | Interventions | - 0PN or 2PN embryos from IVF insemination - Frozen-thawed blastocyst transfer | |  |
|  | Outcomes | Primary outcomes   - Live birth rate: defined as the number of live birth per transfer cycle   Secondary outcomes   - Clinical pregnancy rate: defined as the number of clinical pregnancies per transfer cycle - Miscarriage rate: defined as the number of spontaneous abortion embryos per clinical pregnancy - Malformation rate: defined as the number of malformations per every live birth. - Blastulation rate: defined as the number of blastocysts per cultured embryo | |  |
|  | Notes | - Not all transfers are single embryo transfer, data from double embryo transfers were not included in the meta-analysis | |  |
|  | ***Risk of bias*** | | |  |
| Bias | | ROBINS‐E judgement | Support for judgement | |
| Bias due to confounding | | Some concerns | Embryo transfer outcome   - The following confounders were measured and compared for: participants' age, BMI, and cumulative dose of gonadotropin, and there was no difference found between the 0PN and 2PN groups of patients - The following confounders were measured but not adjusted for: participants' embryo quality - The following confounders were not measured: participants' cause of infertility and the number of previous attempts | |
| Bias arising from measurement of the exposure | | Some concerns | Measurement of exposure was well defined, but there could be error in single time-point assessment | |
| Bias in selection of participants into the study | | Low risk | Participants were not selected based on characteristics observed after the intervention began | |
| Bias due to post-exposure interventions | | Some concerns | Cycles were excluded when there was no cleaved 0PN or 2PN embryos | |
| Bias due to missing data | | No information | No information reported | |
| Bias in measurement of the outcomes | | Some concerns | Blinding of outcome assessors was not reported but outcome measuring method was well defined and standardized. | |
| Bias in selection of the reported result | | Low risk | All measured outcomes were reported | |
| Overall bias | | Some concerns |  | |

Hondo et al., 2019

|  | ***Study characteristics*** | | |  |
| --- | --- | --- | --- | --- |
|  | Methods | - Retrospective study - Single‐centre study at the Denentoshi Ladies Clinic, Kanagawa, Japan - From January 2006 to December 2015 | |  |
|  | Participants | - Age at the time of oocyte retrieval: ≤41 | |  |
|  | Interventions | - 0PN, 1PN or 2PN embryos from IVF or ICSI insemination - Frozen‐thawed single blastocyst transfer | |  |
|  | Outcomes | Primary outcomes   - Live birth rate: defined as the number of live-born per every embryo transferred.   Secondary outcomes   - Clinical pregnancy: defined as the presence of a gestation sac by transvaginal ultrasound - Miscarriage rate: defined as the number of miscarriages per clinical pregnancy - Malformation: defined as the developmental abnormalities during embryonic and fetal periods | |  |
|  | Notes |  | |  |
|  | ***Risk of bias*** | | |  |
| Bias | | ROBINS‐E judgement | Support for judgement | |
| Bias due to confounding | | High risk | Embryo transfer outcome   - Subgroup-analysis was carried out for: fertilisation method - The following confounders were measured but not adjusted for: participants' age - The following confounders were not measured: participants' BMI, cause of infertility, the number of previous attempts, cumulative dose of gonadotropin, and embryo quality | |
| Bias arising from measurement of the exposure | | Some concerns | Measurement of exposure was well defined, but there could be error in single time-point assessment | |
| Bias in selection of participants into the study | | Low risk | Participants were not selected based on characteristics observed after the intervention began | |
| Bias due to post-exposure interventions | | Very high risk | 2PN embryos were prioritized for transfer | |
| Bias due to missing data | | No information | No information reported | |
| Bias in measurement of the outcomes | | High risk | Blinding of outcome assessors was not reported but outcome measuring method was not well defined | |
| Bias in selection of the reported result | | Low risk | All measured outcomes were reported | |
| Overall bias | | High risk |  | |

Labied et al., 2017

|  | ***Study characteristics*** | | |  |
| --- | --- | --- | --- | --- |
|  | Methods | - Retrospective study - Single‐centre study performed at the Center for Reproductive Medicine of the University of Liège, located at the Citadelle Hospital in Belgium - 2762 fresh cycles and 167 frozen–thawed cycles between January 2018 and December 2022 | |  |
|  | Participants | - Age:   Fresh cycle: 36.15 ± 4.6 (IVF); 34.36 ± 5.3 (ICSI)  Frozen–thawed cycle: 36.1 ± 5 (IVF); 34.7 ± 5.8 (ICSI)   - BMI:   Fresh cycle: 24.3 ± 4.1 (IVF); 25.3 ± 5 (ICSI)  Frozen–thawed cycle: 24.6 ± 11.8 (IVF); 25.5 ± 11.9 (ICSI) | |  |
|  | Interventions | - 1PN or 2PN embryos from IVF or ICSI insemination - Fresh or frozen-thawed single blastocyst transfer | |  |
|  | Outcomes | Primary outcomes   - Live birth rate: defined as the number of live-born per every embryo transferred   Secondary outcomes:   - Clinical pregnancy rate: defined as the number of clinical pregnancies per embryo transferred - Miscarriage rate: defined as the number of miscarriages per clinical pregnancy | |  |
|  | Notes |  | |  |
|  | ***Risk of bias*** |  | |  |
| Bias | | ROBINS‐E judgement | Support for judgement | |
| Bias due to confounding | | Low risk | Embryo transfer outcome  Controls were derived from sibling oocytes of the intervention group. | |
| Bias arising from measurement of the exposure | | Some concerns | Measurement of exposure was well defined, but there could be error in single time-point assessment | |
| Bias in selection of participants into the study | | High risk | Only cycles with at least one 1PN zygote were selected | |
| Bias due to post-exposure interventions | | Low risk | Transfer of 1PN/ 2PN embryos was based on morphological and developmental qualities | |
| Bias due to missing data | | No information | No information reported | |
| Bias in measurement of the outcomes | | Low risk | Blinding of outcome assessors was not reported but assessment of the outcome was unlikely to be influenced by knowledge of participants’ exposure history | |
| Bias in selection of the reported result | | Low risk | All measured outcomes were reported | |
| Overall bias | | Some concerns |  | |

Li et al., 2021

|  | ***Study characteristics*** | | |  |
| --- | --- | --- | --- | --- |
|  | Methods | - Retrospective study - Single‐centre study at the Reproductive Medical Center of Peking University Third Hospital - From March 2012 to March 2019 | |  |
|  | Participants | - Exclusion criteria: 1PN zygotes from ICSI insemination, donor-recipient cycles, in vitro maturation cycles, rescue ICSI, artificial oocyte activation cycles, PGT cycles | |  |
|  | Interventions | - 0PN, 1PN or 2PN embryos from IVF or ICSI insemination - Frozen‐thawed single blastocyst transfer | |  |
|  | Outcomes | Primary outcomes   - Live birth rate: defined as the number of live-born per every embryo transferred.   Secondary outcomes   - Clinical pregnancy rate: defined as the number of clinical pregnancies per embryo transferred. - Miscarriage rate: defined as the number of miscarriages per clinical pregnancy | |  |
|  | Notes |  | |  |
|  | ***Risk of bias*** | | |  |
| Bias | | ROBINS‐E judgement | Support for judgement | |
| Bias due to confounding | | Some concerns | Embryo transfer outcome   - The following confounders were measured and adjusted for: participant’s age, BMI, cause of infertility, duration of infertility, number of transfer times, paternal age, fertilisation method, and endometrial preparation - The following confounders were not measured: participants' cumulative dose of gonadotropin, and embryo quality | |
| Bias arising from measurement of the exposure | | Some concerns | Measurement of exposure was well defined, but there could be error in single time-point assessment | |
| Bias in selection of participants into the study | | High risk | 1PN zygotes arising from ICSI were excluded | |
| Bias due to post-exposure interventions | | No information | No information reported | |
| Bias due to missing data | | Low risk | All transfer outcomes were retrieved from electronic medical records | |
| Bias in measurement of the outcomes | | Some concerns | Blinding of outcome assessors was not reported but outcome measuring method was well defined and standardized. | |
| Bias in selection of the reported result | | Low risk | All measured outcomes were reported | |
| Overall bias | | Some concerns |  | |

McCaffrey et al., 2019a

|  | ***Study characteristics*** | | | |  |
| --- | --- | --- | --- | --- | --- |
|  | Methods | | - Retrospective study - Single‐centre study at the NYU Langone Fertility Center - From 2015 to 2018 | |  |
|  | Participants | | - Participants underwent PGT-A | |  |
|  | Interventions | | - Trophectoderm biopsy was performed - PGT-A method was not described - Bi-parental inheritance of 1PN embryo was not investigated - Number of embryo transferred per cycle was not described, thus clinical outcomes of embryo transfer will not be included in this review | |  |
|  | Outcomes | | Secondary outcomes   - Euploidy rate: defined as the number of euploid embryos per embryo biopsied | |  |
|  | Notes | | ASRM abstract only | |  |
|  | ***Risk of bias*** | | | |  |
| Bias | | ROBINS‐E judgement | | Support for judgement | |
| Bias due to confounding | | High risk | | Embryo ploidy outcome   - Stratified analysis was performed on participants' fertilisation method - The following confounders were measured but not adjusted for: participants' embryo quality - The following confounders were not measured: participants' age, BMI, cause of infertility, the number of previous attempts, and cumulative dose of gonadotropin | |
| Bias arising from measurement of the exposure | | Some concerns | | Measurement of exposure was well defined, but there could be error in single time-point assessment | |
| Bias in selection of participants into the study | | No information | | No information reported | |
| Bias due to post-exposure interventions | | Some concerns | | Cases with <50% of the mature eggs exhibiting 2PN were rechecked on Day 1 | |
| Bias due to missing data | | No information | | No information reported | |
| Bias in measurement of the outcomes | | No information | | No information reported | |
| Bias in selection of the reported result | | Low risk | | All measured outcomes were reported | |
| Overall bias | | High risk | |  | |

Tong et al., 2023

|  | ***Study characteristics*** | | | |  |
| --- | --- | --- | --- | --- | --- |
|  | Methods | | - Retrospective study - Single‐centre study at the Sir Run Run Shaw Hospital, affiliated with Zhejiang University School of Medicine - From March 2018 to December 2021 | |  |
|  | Participants | | - All PGT-A cycles underwent ICSI insemination | |  |
|  | Interventions | | - 0PN, 1PN or 2PN embryos from IVF or ICSI insemination - Trophectoderm biopsy was performed on day 5 or 6 - Copy number variation was analyzed using Illumina Nextseq 550 platform (Illumina, San Diego, CA, USA) - Ploidy abnormalities, such as polyploidy, haploidy or uniparental disomy, were detected using Illumina Infinium Asian Screening Array-24 + v1.0 gene chips - Frozen-thawed single blastocyst transfer | |  |
|  | Outcomes | | Primary outcomes   - Live birth: defined as at least one live-born after 28 weeks’ gestation   Secondary outcomes   - Clinical pregnancy rate: defined as the presence of a gestation sac by ultrasound after 5 weeks - Malformation rate: defined as the number of malformations per live birth. - Miscarriage: defined as the loss of a previously visualized intrauterine gestational sac by ultrasound before 20 weeks - Euploidy rate: defined as the number of euploid embryos per embryo biopsied | |  |
|  | Notes | |  | |  |
|  | ***Risk of bias*** | | | |  |
| Bias | | ROBINS‐E judgement | | Support for judgement | |
| Bias due to confounding | | Very high risk | | Embryo ploidy outcome   - The following confounders were not measured: participants' age, BMI, cause of infertility, the number of previous attempts, cumulative dose of gonadotropin, and embryo quality   Embryo transfer outcome   - Stratified analysis was performed on participants' fertilisation method - The following confounders were measured but not adjusted for: participants' embryo quality - The following confounders were not measured: participants' age, BMI, cause of infertility, the number of previous attempts, and cumulative dose of gonadotropin | |
| Bias arising from measurement of the exposure | | Some concerns | | Measurement of exposure was well defined, but there could be error in single time-point assessment | |
| Bias in selection of participants into the study | | Low risk | | Participants were not selected based on characteristics observed after the intervention began | |
| Bias due to post-exposure interventions | | Some concerns | | Cases with <50% of the mature eggs exhibiting 2PN were rechecked on Day 1 | |
| Bias due to missing data | | Some concerns | | Embryo ploidy outcome   - Missing data on 2PN euploidy rate due to amplification failure   Embryo transfer outcome   - Data on pregnancy loss and delivery were acquired through telephone interviews | |
| Bias in measurement of the outcomes | | Some concerns | | Blinding of outcome assessors was not reported but outcome measuring method was well defined and standardized. | |
| Bias in selection of the reported result | | Low risk | | All measured outcomes were reported | |
| Overall bias | | High risk | |  | |

Wozniak et al., 2021

|  | ***Study characteristics*** | | | |  |
| --- | --- | --- | --- | --- | --- |
|  | Methods | - Retrospective study - Single-centre study at Ovation Fertility, Newport Beach, California | | |  |
|  | Participants | - Participants underwent ICSI and PGT-A | | |  |
|  | Interventions | - 0PN, 1PN and 2PN from ICSI insemination - Trophectoderm biopsy was performed on day 5 to 7 - NGS-based PGT-A - DNA from each 1PN embryo’s biological parents were used to assess the ploidy status of 1PN blastocysts without Y chromosome | | |  |
|  | Outcomes | Secondary outcomes   - Blastulation rate: defined as the number of blastocysts formed per every fertilized zygote - Euploidy rate: defined as the number of euploid embryos per embryo biopsied | | |  |
|  | Notes | ASRM abstract only | | |  |
|  | ***Risk of bias*** | | | |  |
| Bias | | | ROBINS‐E judgement | Support for judgement | |
| Bias due to confounding | | | Very high risk | Embryo ploidy outcome   - The following confounders were not measured: participants' age, BMI, cause of infertility, the number of previous attempts, cumulative dose of gonadotropin, and embryo quality | |
| Bias arising from measurement of the exposure | | | Some concerns | Measurement of exposure was well defined, but there could be error in single time-point assessment | |
| Bias in selection of participants into the study | | | No information | No information reported | |
| Bias due to post-exposure interventions | | | No information | No information reported | |
| Bias due to missing data | | | No information | No information reported | |
| Bias in measurement of the outcomes | | | Some concerns | Blinding of outcome assessors was not reported but outcome measuring method was well defined and standardized. | |
| Bias in selection of the reported result | | | Low risk | All measured outcomes were reported | |
| Overall bias | | | High risk |  | |

Xu et al., 2025

|  | ***Study characteristics*** | | | |  |
| --- | --- | --- | --- | --- | --- |
|  | Methods | | - Retrospective study - Single‐centre study at the Shanghai Ninth People’s Hospital - From January 2012 to April 2021 | |  |
|  | Participants | | - Age (median [25^th^ percentile, 75^th^ percentile]):   33 [30, 36] (0PN); 32 [30, 35] (1PN); 33 [30, 36] (2PN)   - BMI (median [25^th^ percentile, 75^th^ percentile]):   22.03 [20.19, 24.05] (0PN); 21.23 [19.57, 23.44] (1PN); 21.38 [19.57, 23.44] (2PN)   - Exclusion criteria: 1PN zygotes derived from ICSI cycles, sperm/ oocyte donation cycles, in vitro maturation cycles, rescue ICSI, artificial oocyte activation cycles, women with preexisting diabetes and hypertension before pregnancy, loss to follow-up cases | |  |
|  | Interventions | | - 0PN or 2PN embryos from IVF or ICSI insemination or 1PN embryos from IVF insemination - Frozen-thawed single blastocyst transfer | |  |
|  | Outcomes | | Primary outcomes   - Live birth: defined as a live neonate born after 20 weeks’ gestation   Secondary outcomes   - Clinical pregnancy: confirmed by the presence of at least 1 gestational sac in the uterine cavity on ultrasound scanning 5 weeks after embryo transfer - Miscarriage: defined as the loss of a spontaneous clinical pregnancy before 20 weeks of gestation - Malformation rate: Congenital malformations were defined according to the International Statistical Classification of Diseases and Related Health Problems-10th Revision (ICD-10) | |  |
|  | Notes | |  | |  |
|  | ***Risk of bias*** | | | |  |
| Bias | | ROBINS‐E judgement | | Support for judgement | |
| Bias due to confounding | | Low risk | | Embryo transfer outcome  Analyses were adjusted for age, body mass index, infertility duration, parity, main infertility cause, frozen embryo transfer cycle rank, insemination method, embryo quality, endometrial preparation, and year of treatment | |
| Bias arising from measurement of the exposure | | Some concerns | | Measurement of exposure was well defined, but there could be error in single time-point assessment | |
| Bias in selection of participants into the study | | High risk | | 1PN zygotes derived from ICSI cycles were excluded | |
| Bias due to post-exposure interventions | | No information | | No information reported | |
| Bias due to missing data | | Some concerns | | Loss to follow-up cases were excluded | |
| Bias in measurement of the outcomes | | Some  concerns | | Blinding of outcome assessors was not reported but outcome measuring method was well defined and standardized. | |
| Bias in selection of the reported result | | Low risk | | All measured outcomes were reported | |
| Overall bias | | Some concerns | |  | |

Zhu et al., 2024b

|  | | ***Study characteristics*** | | | |  |
| --- | --- | --- | --- | --- | --- | --- |
|  | | Methods | - Retrospective study - Single‐centre study at the Huzhou Maternity and Child Health Care Hospital in China - From January 2020 to December 2021 | | |  |
|  | | Participants | - Mean age (SD) of single blastocyst transferred cycles: 0PN 32.3 (5.8), 1PN 32.0 (6.1), 2PN 30.1 (4.3) - Inclusion criteria: patients aged 20 to 45 years - Exclusion criteria: ICSI cycles and loss to follow-up cases | | |  |
|  | | Interventions | - 0PN, 1PN and 2PN embryos from IVF insemination - Single or double blastocyst transfer | | |  |
|  | | Outcomes | Primary outcomes   - Live birth: defined as delivery of a delivery of a living infant at ≥ 22nd weeks of gestation   Secondary outcomes   - Clinical pregnancy: defined as the observation of a gestational sac under ultrasound examination on day 35 after embryo transfer - Miscarrage: miscarriage before the 22nd gestational week - Malformation: not defined - Blastulation rate: defined as the number of blastocysts formed per fertilized zygote | | |  |
|  | | Notes | Not all transfers are single embryo transfer, data from double embryo transfers were not included in the meta-analysis. | | |  |
|  | ***Risk of bias*** | | | | |  |
| Bias | | | | ROBINS‐E judgement | Support for judgement | |
| Bias due to confounding | | | | High risk | Embryo transfer outcome  • The following confounders were measured but not adjusted for: participants' age  • The following confounders were not measured: participants' BMI, cause of infertility, the number of previous attempts, cumulative dose of gonadotropin, and embryo quality | |
| Bias arising from measurement of the exposure | | | | Some concerns | Measurement of exposure was well defined, but there could be error in single time-point assessment | |
| Bias in selection of participants into the study | | | | Low risk | Participants were not selected based on characteristics observed after the intervention began | |
| Bias due to post-exposure interventions | | | | High risk | 2PN embryos were give higher priority for transfer | |
| Bias due to missing data | | | | Some concerns | Loss to follow-up cases were excluded | |
| Bias in measurement of the outcomes | | | | Some  concerns | Blinding of outcome assessors was not reported but outcome measuring method was well defined and standardized. | |
| Bias in selection of the reported result | | | | Low risk | All measured outcomes were reported | |
| Overall bias | | | | Some concerns |  | |

Supplementary Table S1. Characteristics of excluded studies [ordered by study ID]

| **Study** | **Reason for exclusion** |
| --- | --- |
| Al Hashimi et al., 2024 | PN observation time not specified |
| Al Hashimi et al., 2025 | Raw data not available for analysis |
| Aoi et al., 2010 | Not single embryo transfer |
| Barberi et al., 2025 | Raw data not available for analysis |
| Brinkmann et al., 2021 | PN observation time not specified |
| Cagnin et al., 2023 | PN observation time not specified |
| Campos et al., 2007 | PN observation time out of range |
| Capalbo et al., 2017a | The study did not include a control group |
| Capalbo et al., 2017b | The study did not include a control group |
| Catala et al., 2017 | The study did not include a control group |
| Chen et al., 2022 | Duplicated publication |
| Curnow et al., 2023 | PN observation time out of range |
| Destouni et al., 2018 | PN observation time out of range |
| Feferkorn et al., 2022 | PN observation time not specified |
| Freitas et al., 2020 | PN observation time out of range |
| Fu et al., 2022 | Not single embryo transfer |
| Girardi et al., 2023 | PN observation time not specified |
| Girardi et al., 2025 | PN observation time not specified |
| Gomez Pena et al., 2022 | PN observation time out of range |
| Grkovic et al., 2019 | PN observation time not specified |
| Heath et al., 2012 | PN observation time out of range |
| Henry et al., 2021 | PN observation time not specified |
| Henry et al., 2023a | PN observation time not specified |
| Henry et al., 2023b | PN observation time not specified |
| Hirata et al., 2020 | PN observation time out of range |
| Itoi et al., 2015 | Not single embryo transfer |
| Jordan et al., 2022 | PN observation time not specified |
| Kai et al., 2018 | PN observation time out of range |
| Kayisli et al., 2010 | Cleavage stage embryo biopsy |
| Kim et al., 2013 | PN observation time not specified |
| Kobayashi et al., 2021 | PN observation time out of range |
| Lebovitz et al., 2024 | Cleavage stage embryo biopsy |
| Lee et al., 2013 | PN observation time out of range |
| Li et al., 2015a | PN observation time out of range |
| Li et al., 2015b | PN observation time out of range |
| Li et al., 2020 | PN observation time out of range |
| Liao et al., 2009 | The study did not include a control group |
| Liu et al., 2016 | PN observation time out of range |
| Ma et al., 2018 | Not single embryo transfer |
| Mateo et al., 2013 | PN observation time out of range |
| Mateo et al., 2017 | PN observation time not specified |
| McCaffrey et al., 2019b | Duplicated publication |
| McCallie et al., 2024 | The study did not include a control group |
| Mizuta et al., 2011 | PN observation time not specified |
| Mizuta et al., 2018 | PN observation time not specified |
| Mizuta et al., 2019 | PN observation time not specified |
| Moon et al., 2025 | PN observation time out of range |
| Nohales et al., 2018 | PN observation time not specified |
| Noyes et al., 2007 | The study did not include a control group |
| Noyes et al., 2008 | Cleavage stage embryo biopsy |
| Otsu et al., 2004 | PN observation time out of range |
| Paun et al., 2025 | Raw data not available for analysis |
| Paz et al., 2020 | PN observation time out of range |
| Perry et al., 2023 | PN observation time not specified |
| Perry et al., 2025 | Raw data not available for analysis |
| Picou et al., 2019 | PN observation time out of range |
| Potts et al., 2017 | PN observation time not specified |
| Rao et al., 2020 | Not single embryo transfer |
| Romanelli et al. 2019 | PN observation time not specified |
| Sayers et al., 2011 | PN observation time out of range |
| Seckin et al., 2023 | PN observation time not specified |
| Shimizu et al., 2017 | PN observation time not specified |
| Shun et al., 2023 | PN observation time not specified |
| Si et al., 2019 | Duplicated publication |
| Soler et al., 2021 | PN observation time out of range |
| Staessen & Van Steirteghem, 1997 | PN observation time not specified |
| Stock-Myer et al., 2025 | The study did not include a control group |
| Tan et al., 2014 | PN observation time not specified |
| Tsuji et al., 2016 | PN observation time not specified |
| Tsuji et al., 2020 | PN observation time not specified |
| Ussher et al., 2023 | The study did not include a control group |
| Wang et al., 2022 | Duplicated publication |
| Wang et al., 2025 | PN observation time out of range |
| Wei et al., 2019 | Not single embryo transfer |
| Wen et al., 2024 | PN observation time not specified |
| Werlin et al., 2007 | PN observation time not specified |
| Wozniak et al., 2020 | Duplicated publication |
| Xie et al., 2018 | The study did not include a control group |
| Xiong et al., 2024 | PN observation time out of range |
| Xu et al., 2015 | PN observation time not specified |
| Yao et al., 2016 | PN observation time out of range |
| Yelke et al., 2023 | PN observation time not specified |
| Yeshua et al., 2024 | PN observation time not specified |
| Zhang et al., 2019 | Not single embryo transfer |
| Zhang et al., 2023 | PN observation time out of range |
| Zhao et al., 2022 | PN observation time not specified |
| Zhu et al., 2024a | PN observation time out of range |

PN: pronuclei

Supplementary Figure S1. Funnel plots comparing outcomes between 0PN and 2PN embryos

| a) Live birth rate between 0PN embryos and 2PN embryos    | b) Clinical preganancy rate between 0PN embryos and 2PN embryos    |
| --- | --- |
| c) Miscarriage rate between 0PN embryos and 2PN embryos    | d) Malformation rate between 0PN embryos and 2PN embryos    |
| e) Blastulation rate between 0PN embryos and 2PN embryos    | f) Euploidy rate between 0PN embryos and 2PN embryos    |

**Supplementary Figure S1.** Funnel plots comparing outcomes between 0PN and 2PN embryos. Funnel plots of a) live birth rate, b) clinical pregnancy rate, c) miscarriage rate, d) malformation rate, e) blastulation rate and f) euploidy rate between nonpronuclear (0PN) embryos and dipronuclear (2PN) embryos.

Supplementary Figure S2. Funnel plots comparing outcomes between 1PN and 2PN embryos

| a) Live birth rate between 1PN embryos and 2PN embryos    | b) Clinical preganancy rate between 1PN embryos and 2PN embryos    |
| --- | --- |
| c) Miscarriage rate between 1PN embryos and 2PN embryos    | d) Malformation rate between 1PN embryos and 2PN embryos    |
| e) Blastulation rate between 1PN embryos and 2PN embryos    | f) Euploidy rate between 1PN embryos and 2PN embryos    |

**Supplementary Figure S2.** Funnel plots comparing outcomes between 1PN and 2PN embryos. Funnel plots of a) live birth rate, b) clinical pregnancy rate, c) miscarriage rate, d) malformation rate, e) blastulation rate and f) euploidy rate between monopronuclear (1PN) embryos and dipronuclear (2PN) embryos.

Supplementary Data File S3. Subgroup analyses for euploidy rate

Supplementary Data File S3 - Figure 1. Forest plots comparing euploidy rates between 0PN embryos and 2PN embryos stratified by insemination method (IVF vs. ICSI). Each plot presents the odds ratios (ORs) with 95% confidence intervals (CIs) for individual studies, with pooled estimates calculated using a random-effects model. Horizontal lines represent individual study estimates, and diamonds represent overall effects. Heterogeneity was assessed using the *I*² statistic.

Supplementary Data File S3 - Figure 2. Forest plots comparing euploidy rates between 1PN embryos and 2PN embryos (a) with and (b) without bi-parental inheritance confirmation for 1PN embryos, stratified by insemination method (IVF vs. ICSI). Each plot presents the odds ratios (ORs) with 95% confidence intervals (CIs) for individual studies, with pooled estimates calculated using a random-effects model. Horizontal lines represent individual study estimates, and diamonds represent overall effects. Heterogeneity was assessed using the *I*² statistic.

**Outcome comparison between 0PN embryos versus 2PN embryos**

Euploidy rate

*IVF subgroup*

Only one of the included studies reported euploidy rates of 0PN embryos inseminated by IVF (McCaffrey et al., 2019a) and showed that 0PN embryos inseminated by IVF has a significantly lower euploidy rate than that of 2PN embryos (Figure 3f; OR = 0.60, 95% CI = 0.41-0.88, p-value = 0.009; 1 study, N = 11,434; I² = N.A.).

*ICSI subgroup*

Three studies reported euploidy rates of 0PN embryos inseminated by ICSI (McCaffrey et al., 2019a; Tong et al., 2023; Wozniak et al., 2021) and showed no significant difference between 0PN and 2PN embryos on euploidy rate (Figure 3f; OR = 1.16, 95% CI = 0.78-1.72, p-value = 0.46; 3 studies, N = 10,275; I² = 0%).

**Outcome comparison between 1PN embryos versus 2PN embryos**

Euploidy rate

*IVF subgroup*

Two studies reported euploidy rate of 1PN embryos inseminated by IVF (Bradley et al., 2017; McCaffrey et al., 2019a). There was a significant difference between 1PN and 2PN embryos on euploidy rate in IVF with results favouring 2PN embryos (Figure 5f; OR 0.73, 95% CI = 0.59-0.90, p-value = 0.003; 2 studies, N = 13,906; I² = 5%).

*ICSI subgroup*

Four studies reported euploidy rates of 1PN embryos (Bradley et al., 2017; McCaffrey et al., 2019a; Tong et al., 2023; Wozniak et al., 2021). There was no significant difference between 1PN and 2PN embryos on euploidy rate (Figure 5f; OR = 1.31, 95% CI = 0.94-1.85, p-value = 0.12; 4 studies, N = 13,034; I² = 7%).

Euploidy rate with bi-parental inheritance confirmation for 1PN embryos

*IVF subgroup*

Only one of the 11 included studies reported bi-parental inheritance confirmation of 1PN embryos inseminated by IVF (Bradley et al., 2017). The rate of 1PN embryos that were euploid and with confirmed bi-parental inheritance was not significantly different from the euploidy rate of 2PN embryos (Figure 5g; OR = 0.90, 95% CI = 0.57-1.44, p-value = 0.67; 1 study, N = 2,191; I² = N.A.).

*ICSI subgroup*

Three studies reported bi-parental inheritance confirmation of 1PN embryos inseminated by ICSI (Bradley et al., 2017; Tong et al., 2023; Wozniak et al., 2021). The rate of 1PN embryos that were euploid and with confirmed bi-parental inheritance was not significantly different from the euploidy rate of 2PN embryos (Figure 5g; OR = 0.83, 95% CI = 0.54-1.29, p-value = 0.41; 3 studies, N = 6,405; I² = 0%).

Supplementary Figure S3. Sensitivity analyses

# a) Miscarriage rate (0PN vs 2PN)

# b) Malformation rate (0PN vs 2PN)

# c) Miscarriage rate (1PN vs 2PN)

# d) Malformation rate (1PN vs 2PN)

**Supplementary Figure S3.** Sensitivity analyses using events per embryo transfer for miscarriage and malformation rates showed no change in the conclusions. Forest plots of a) miscarriage rate and b) malformation rate comparing nonpronuclear (0PN) embryos with dipronuclear (2PN) embryos, and c) miscarriage rate and d) malformation rate comparing monopronuclear (1PN) embryos with 2PN embryos.

Supplementary Table S2. Summary of adjusted odds ratios from Li et al. (2021)

|  | | 0PN vs 2PN | 1PN vs 2PN |
| --- | --- | --- | --- |
| Live Birth Rate | Unadjusted OR | 1.022 (0.837–1.248) | 0.697 (0.535–0.908) |
|  | Adjusted OR | 1.056 (0.833–1.338) | 0.701 (0.496–0.992) |
| Clinical Pregnancy Rate | Unadjusted OR | 0.973 (0.802–1.177) | 0.818 (0.643–1.039) |
|  | Adjusted OR | 1.012 (0.806–1.270) | 0.792 (0.580–1.082) |
| Miscarriage Rate | Unadjusted OR | 0.914 (0.64–1.305) | 1.668 (1.111–2.505) |
|  | Adjusted OR | 1.003 (0.616–1.546) | 1.764 (1.065–2.974) |

**Supplementary Table S2.** Summary of adjusted odds ratios from Li et al. (2021). Logistic regression analyses accounting for maternal age, maternal BMI, type of infertility, duration of infertility, number of transfer times, paternal age, cause of infertility, fertilization method, and vitrified-warmed transfer endometrial preparation, as reported in Li et al. (2021).

Supplementary Table S3. Summary of adjusted odds ratios from Xu et al. (2025)

|  | | 0PN vs 2PN | 1PN vs 2PN |
| --- | --- | --- | --- |
| Live Birth Rate | Unadjusted OR | 1.06 (0.86-1.31) | 0.99 (0.79-1.26) |
|  | Adjusted OR | 1.09 (0.88-1.36) | 0.99 (0.78-1.26) |
| Clinical Pregnancy Rate | Unadjusted OR | 1.11 (0.90-1.36) | 0.95 (0.75-1.19) |
|  | Adjusted OR | 1.13 (0.92-1.40) | 0.93 (0.73-1.17) |
| Miscarriage Rate | Unadjusted OR | 1.03 (0.70-1.52) | 0.80 (0.49-1.29) |
|  | Adjusted OR | 0.96 (0.64-1.43) | 0.76 (0.47-1.25) |

**Supplementary Table S3.** Summary of adjusted odds ratios from Xu et al. (2025). Multivariable regression analyses for age, body mass index, infertility duration, parity, main infertility cause, frozen embryo transfer cycle rank, insemination method, embryo quality, endometrial preparation, and year of treatment, as reported in Xu et al. (2025).

References

Al Hashimi B, Linara-Demakakou E, Green O, Raikundalia B, Shah T, Griffin D, Ahuja K, Macklon N. O-035 Euploidy’s hidden potential: emerging insights from abnormal fertilized embryos. *Human Reproduction* 2024;**39(Supplement_1)**:i19

Al Hashimi B, Harvey SC, Harvey KE, Linara-Demakakou E, Raikundalia B, Green O, Griffin DK, Ahuja K, Macklon N. Reassessing the conventional fertilization check: leveraging preimplantation genetic testing for aneuploidy to increase the number of transferrable embryos. *Reproductive BioMedicine Online* 2025;**50(6):**104595.

Aoi Y, Takahashi H, Saitou H, Takiue C, Kawakami N, Tone M, Hirata R, Terada S, Yoshioka N, Habara T et al. Development potential and clinical implication of embryos having either one pronucleus, (1PN) or no pronuclei (0PN). *Human Reproduction* 2010;**25:**I184-5.

Barberi M, Ruberti A, Pirastu G, Biricik A, Listorti I, Greco P, Greco E. O-103 Incidence, development and ploidy of abnormally fertilized oocyte: implications for clinical practice. *Human Reproduction* 2025;**40(Supplement_1)**:i62.

Bradley CK, Traversa MV, Hobson N, Gee AJ, McArthur SJ. Clinical use of monopronucleated zygotes following blastocyst culture and preimplantation genetic screening, including verification of biparental chromosome inheritance. *Reproductive BioMedicine Online* 2017;34(6):567-574.

Brinkmann E, Demmers Werken C, Ramos L. Monopronuclear (1PN) embryos can derive in healthy pregnancies. Human Reproduction 2021;36(SUPPL 1):i196-7.

Cagnin N, De Souza MU, Estrada PR, Souza CC, Miravet-Valenciano J, Lluesa CR, Coprerski B, Riboldi M, Valdez F. DETERMINATION OF PLOIDY LEVEL INTEGRATED INTO THE COMMON PREIMPLANTATION GENETIC TESTING FOR ANEUPLOIDY (PGT-A) AND ASSOCIATION WITH THE PRONUCLEI NUMBER. *Fertility and Sterility* 2023;**120(4)**;e2.

Campos G, Parriego M, Vidal F, Coroleu B, Veiga A. Cytogenetic constitution and developmental potential of embryos derived from apronuclear and monopronuclear zygotes. *Revista Iberoamericana de Fertilidad y Reproduccion Humana* 2007;**24(1)**:29-34.

Capalbo A, Treff N, Cimadomo D, Tao X, Ferrero S, Vaiarelli A, Colamaria S, Giuliani M, Sapienza F, Ubaldi FM et al. Blastocysts from abnormally-fertilized zygotes can be euploid/diploid and are reproductively competent: live-births from preimplantation genetic diagnosis of aneuploidy/polyploidy in embryos with abnormal pronuclear morphology. *Human Reproduction* 2017;**32**:243.

Capalbo A, Treff N, Cimadomo D, Tao X, Ferrero S, Vaiarelli A, Colamaria S, Maggiulli R, Orlando G, Scarica C et al. Abnormally fertilized oocytes can result in healthy live births: improved genetic technologies for preimplantation genetic testing can be used to rescue viable embryos in in vitro fertilization cycles. *Fertility and sterility* 2017;**108(6)**:1007-1015.

Catala MK, Sieren KR, Silverberg K, VerMilyea MD. The value in retaining non-pronuclear (0PN) zygotes for extended culture. *Fertility and Sterility* 2017;**108(3)**:e150-151.

Chen C, Li W, Yin M, Li M, Wu L, Si J, Zhao L, Li B, Yan Z, Lyu Q. Does the cell number of 0PN embryos on day 3 affect pregnancy and neonatal outcomes following single blastocyst transfer?. *BMC Pregnancy and Childbirth* 2022;**22(1)**:200.

Chen X, Shi S, Mao J, Zou L, Yu K. Developmental potential of abnormally fertilized oocytes and the associated clinical outcomes. Frontiers in Physiology 2020;11:528424.

Curnow EC, Sullivan AE, Herndon CN, Ryan GL. NON-PRONUCLEAR ZYGOTES (0PN): AN UNDERAPPRECIATED SOURCE OF GENETICALLY NORMAL BLASTOCYSTS. *Fertility and Sterility* 2023;**120(1)**:e25-6.

Destouni A, Dimitriadou E, Masset H, Debrock S, Melotte C, Van Den Bogaert K, Zamani Esteki M, Ding J, Voet T, Denayer E et al. Genome-wide haplotyping embryos developing from 0PN and 1PN zygotes increases transferrable embryos in PGT-M. *Human Reproduction* 2018;**33(12):**2302-11.

Feferkorn I, Albar M, Suarthana E, Ao A, Zhang XY, Zhang L, Hizkiyahu R, Liu KE, Buckett WM, Raina J. THE BLASTULATION RATE AND THE RATIO OF 1PN/2PN EMBRYOS ARE ASSOCIATED WITH THE COHORT OF EMBRYOS PLOIDY RATIO. *Fertility and Sterility* 2022;**118(4)**:e251.

Freitas D, Teixeira C, Almeida A, Queiroz L, Carvalho R, Oliveira T, Santos C, Abreu T, Niag M, Coelho G. Embryonic development and cytogenetic evaluation of apronuclear zygotes after 18 to 20 hours of in vitro fertilization. *Human Reproduction* 2020;**35**:I248.

Fu L, Chu D, Zhou W, Li Y. Strictly selected Mono-and non-pronuclear blastocysts could result in appreciable clinical outcomes in IVF cycles. *Human Fertility* 2022;**25(3)**:470-7.

Fu L, Zhou W, Li Y. Development and frozen-thawed transfer of non-pronuclear zygotes-derived embryos in IVF cycles. European Journal of Obstetrics & Gynecology and Reproductive Biology 2021;264:206-211.

Girardi L, Cogo F, Zambon P, Pergher I, Castellòn JA, Patassini C, Rubio C. O-209 The incidence of different ploidy alterations in abnormally fertilized oocytes (AFO)-derived embryos. *Human Reproduction* 2023;**38(Supplement_1)**:i130.

Girardi L, Al Asmar Piñar N, Campos-Galindo I, Castellon J, Akinwole A, Kayali R, Patassini C, Coprerski B, Rubio C, Rodrigo L. P-598 Impact of female age on the ploidy status of preimplantation embryos according to their pronuclear configuration. *Human Reproduction* 2025;**40(Supplement_1)**:i494.

Gomez Peña M, Papayannis M, De Martino E, Filardi P, Magri L, Chiarello JM, Figueroa C, De Zuñiga I, Kopcow L, Horton M et al. O-077 The chromosomal constitution of embryos developing from 0PN zygotes. *Human Reproduction* 2022;37**(Supplement_1)**:i46.

Grkovic S, Bui D, McArthur S. Impact of technology changes on the identification and testing of abnormally fertilized embryos. *Reproductive BioMedicine Online* 2019;**38**:e53-4.

Heath C, Doshi A, Cawood SR, Sabhnani T, Sarna U, Serhal P. Clinical pregnancies and live births achieved from the transfer of 0PN embryos post-intracytoplasmic sperm injection. *Human Fertility* 2012;**15(SUPPL. 1)**:10.

Henry L, Desmecht A, Wenders F, Gaspard O, Labied S, Ravet S, Nisolle M. P-245 Birth of twenty-seven healthy babies following transfer of fresh and frozen-thawed embryos derived from monopronuclear zygotes: a retrospective study. *Human Reproduction* 2023;**38(Supplement_1)**:i306.

Henry L, Haywood ME, Lee R, McCallie BR, Schoolcraft WB, Katz-Jaffe M. PRONUCLEAR NUMERATION DOES NOT ACCURATELY REFLECT THE TRUE NUMBER OF PARENTAL GENOMES. *Fertility and Sterility* 2023;**120(4)**:e102-3.

Henry L, Haywood ME, McReynolds S, McCallie BR, Schoolcraft WB, Katz-Jaffe MG. PRONUCLEAR NUMERATION SHOULD NOT BE THE ONLY METHOD TO DETERMINE ABNORMAL FERTILIZATION IN ORDER TO ACCOUNT FOR ALL EUPLOID EMBRYOS. *Fertility and Sterility* 2021;116(3):e166.

Hirata K, Goto S, Izumi Y, Taguchi M, Hayashi A, Fujioka M, Ishiko A, Nakanishi K, Kimura F, Murakami T. Chromosome analysis of blastocysts derived from single pronuclear zygotes by array CGH and clinical outcomes by the transfer of single pronuclear zygotes. *Journal of Assisted Reproduction and Genetics* 2020;**37**:1645-52.

Hondo S, Arichi A, Muramatsu H, Omura N, Ito K, Komine H, Monzen S, Mukai N, Endo M, Katase S. Clinical outcomes of transfer of frozen and thawed single blastocysts derived from nonpronuclear and monopronuclear zygotes. *Reproductive Medicine and Biology* 2019;**18(3)**:278-283./

Itoi F, Asano Y, Shimizu M, Honnma H, Murata Y. Birth of nine normal healthy babies following transfer of blastocysts derived from human single-pronucleate zygotes. *Journal of assisted reproduction and genetics* 2015;**32**:1401-7.

Jordan A, Toh E, Nguyen K, Venier B. TESTING AND TRANSFER OF 0PN AND 1PN EMBRYOS: WHAT CAN WE LEARN?. *Fertility and Sterility* 2022;**118(5)**:e15.

Kai Y, Moriwaki H, Yumoto K, Iwata K, Mio Y. Assessment of developmental potential of human single pronucleated zygotes derived from conventional in vitro fertilization. *Journal of Assisted Reproduction and Genetics* 2018;**35**:1377-84.

Kayisli UA, Tabak MS, Gerasimova T, Lalioti M, Sakkas D, Patrizio P. What to do with embryos originating from one pronucleus or initial no fertilization?. *Fertility and Sterility* 2010;**94(4)**:S36.

Kim MJ, Cho JW, Shin SB, Kang IS, Lee HS. The atypical zygotes of abnormal pronuclear and embryo quality from intracytoplasmic sperm injection (ICSI) may predict the risk of chromosome abnormality. *Fertility and Sterility* 2013;**100(3)**:S205.

Kobayashi T, Ishikawa H, Ishii K, Sato A, Nakamura N, Saito Y, Hasegawa H, Fujita M, Mitsuhashi A, Shozu M. Time-lapse monitoring of fertilized human oocytes focused on the incidence of 0PN embryos in conventional in vitro fertilization cycles. *Scientific reports* 2021;**11(1)**:18862.

Labied S, Wenders F, Gaspard O, Ravet S, Desmecht A, Nisolle M, Henry L. Birth of Thirty-Two Healthy Babies Following Transfer of Fresh and Frozen–Thawed Embryos Derived from Monopronuclear Zygotes: A Retrospective Study. *Medicina* 2024;**60(8)**:1361.

Lebovitz O, Noach-Hirsh M, Taieb S, Haas J, Zilberberg E, Nahum R, Orvieto R, Aizer A. Embryos derived from single pronucleus are suitable for preimplantation genetic testing. *Fertility and Sterility* 2024;**122(4)**:598-606.

Lee CS, Yap WY, Low SY, Lim YX. P-23 euploidy rates for day 3 apronuclear (0PN) and unipronuclear (1PN) embryos. *Reproductive BioMedicine Online* 2013;**26**:S36.

Li M, Dang Y, Wang Y, Li J, Liu P. Value of transferring embryos derived from monopronucleated (1PN) zygotes at the time of fertilization assessment. *Zygote* 2020;28(3):241-6.

Li M, Huang J, Zhuang X, Lin S, Dang Y, Wang Y, Liu D, Li R, Liu P, Qiao J. Obstetric and neonatal outcomes after the transfer of vitrified-warmed blastocysts developing from nonpronuclear and monopronuclear zygotes: a retrospective cohort study. *Fertility and sterility* 2021;115(1):110-117.

Li M, Lin S, Chen Y, Zhu J, Liu P, Qiao J. Value of transferring embryos that show no evidence of fertilization at the time of fertilization assessment. *Fertility and sterility* 2015;**104(3)**:607-11.

Li M, Liu P, Qiao J. Value of transferring embryos formed by non-pronuclear oocytes at the time of fertilization assessment. *Human Reproduction* 2015;**30**:200.

Liao H, Zhang S, Cheng D, OuYang Q, Lin G, Gu Y, Lu C, Gong F, Lu G. Cytogenetic analysis of human embryos and embryonic stem cells derived from monopronuclear zygotes. *Journal of assisted reproduction and genetics* 2009;**26**:583-589.

Liu J, Wang XL, Zhang X, Shen CY, Zhang Z. Live births resulting from 0PN-derived embryos in conventional IVF cycles. *Journal of assisted reproduction and genetics* 2016;**33**:373-8.

Ma SY, Li C, Zhao HB, Zhang JY, Zhang HZ, Wu KL, Li M. Comparison of clinical outcomes of frozen-thawed blastocysts derived from non-pronucleus or two pronucleus zygotes. *Zhonghua fu Chan ke za zhi* 2018;**53(11)**:749-54.

Mateo S, Parriego M, Boada M, Vidal F, Coroleu B, Veiga A. In vitro development and chromosome constitution of embryos derived from monopronucleated zygotes after intracytoplasmic sperm injection. *Fertility and sterility* 2013;**99(3)**:897-902.

Mateo S, Vidal F, Parriego M, Rodriguez I, Montalvo V, Veiga A, Boada M. Could monopronucleated ICSI zygotes be considered for transfer? Analysis through time-lapse monitoring and PGS. *Journal of Assisted Reproduction and Genetics* 2017;**34**:905-11.

McCaffrey C, McCulloh DH, Lee HL, Besser AG, He X, Licciardi FL, Grifo JA. Euploid embryos whereâ only 1PN OR no pronuclei (PN) Were seen have delivery rates comparable to euploid 2PN embryos. *Fertility and Sterility* 2019;**112(3)**:e424-425.

McCaffrey C, McCulloh DH, Licciardi FL, Grifo JA, Lee HL, He X, Besser AG. How important is it to visualize 2PN in zygotes destined for PGT-A testing by next generation sequencing (NGS)?. *Fertility and Sterility* 2019;**112(3)**:e232.

McCallie BR, Haywood ME, Henry LN, Lee RM, Schoolcraft WB, Katz-Jaffe MG. The Identification of Molecular Ploidy Status of Abnormal Pronuclear Zygotes Reveals a Significant Number of Euploid Blastocysts Available for Conception. *Biomedicines* 2024;**13(1)**:51.

Mizuta S, Hashimoto H, Kuroda Y, Matsumoto Y, Mizusawa Y, Ogata S, Yamada S, Kokeguchi S, Noda Y, Shiotani M. Chromosomal analysis of blastocysts developed from monopronuclear embryos after IVF and ICSI. *Human Reproduction* 2011;**26**:I197-8.

Mizuta S, Matsubayashi H, Takeuchi T, Tamura Y, Santo M, Kitaya K, Araki Y, Ishikawa T. Cytogenetic analysis by next generation sequencing disclosed that extremely high euploidy rate of blastocysts derived from monopronuclear embryos with testicular sperm. *Fertility and Sterility* 2019;**112(3)**:e203.

Mizuta S, Matsubayashi H, Tamura Y, Santo M, Araki Y, Takeuchi T, Kitaya K, Ishikawa T. Monopronuclear embryos derived from testicular sperm extraction followed by intra cytoplasmic sperm injection revealed extremely high euploidy rate with next generation sequencing. *Human Reproduction* 2018;**33**:412-3.

Moon JH, Zhang J, Yu B. Developmental potential of zygotes without pronucleus (0PN) and pregnancy outcomes after single euploid blastocyst transfers. *Journal of Assisted* *Reproduction and Genetics* 2025;**42(4)**:1309

Nohales M, Soler N, Gonzalez-Picazo A, Escriba M, Vendrell X, Bautista-Llacer R, Tena R, Pabon D. Ploidy condition and developmental competence of unipronuclear embryos. *Fertility and Sterility* 2018;**110(4)**:e191.

Noyes N, Fino ME, Krey L, McCaffrey C, Adler A, Grifo J. Embryo biopsy: the fate of abnormal pronuclear embryos. *Reproductive biomedicine online* 2008;**17(6)**:782-788.

Noyes N, Fino E, McCaffrey C, Adler A, Krey L, Grifo J. Embryo biopsy the fate of biopsied abnormal pronuclear embryos. *Fertility and Sterility* 2007;**88**:S29-30.

Otsu E, Sato A, Nagaki M, Araki Y, Utsunomiya T. Developmental potential and chromosomal constitution of embryos derived from larger single pronuclei of human zygotes used in in vitro fertilization. *Fertility and Sterility* 2004;**81(3)**:723-4.

Paun O, Thomson A, Coffey H, Bailey M, Ruso H, Hickman C, Popa T, Iliev D, Taneja J, AlChami A. P-595 Rethinking Fertilisation Assessment: PGT-A Identifies Diploid Embryos Among Morphologically Abnormal Zygotes and Reveals Undetected Polyploidy in 2PN Embryos. *Human Reproduction* 2025;**40(Supplement_1)**:i492.

Paz MV, Chiera M, Hovanyecz P, Cicaré J, Perfumo P, Domenech L, Ventura V. Blastocysts derived from 0PN oocytes: Genetic and clinical results. *JBRA Assisted Reproduction* 2020;**24(2)**:143.

Perry S, Wells D, Batha S, Babariya D, Moore C, Nicopoullos J. O-181 Utilisation of monopronucleated (1PN) embryos and potential clinical benefits for patients. *Human Reproduction* 2023;**38(Supplement 1)**:i111-2.

Perry S, Nicopoullos J, Batha S, Wells D, Glynn K, Babariya D, Clark G. P-273 Blastocysts derived from monopronucleated zygotes are frequently diploid, have potential to produce viable pregnancies, and should not be discarded unless genetic abnormality is confirmed. *Human Reproduction* 2025;**40(Supplement_1)**:i332.

Picou A, Werland H, Staha A, Silverberg K, VerMilyea M. Keep or toss? A retrospective evaluation of the benefits from culturing 0PN 1PB embryos zygotes after∼ 18hrs post ICSI. *Reproductive* *BioMedicine Online* 2019;**39**:e4.

Potts C, Gordon JD, DiMattina M, Fiorentino A, Celia GF. Implantation and live births following transfer of 0pn embryos in natural cycle IVF. *Fertility and Sterility* 2017;**108(3)**:e332.

Rao J, Qiu F, Tian S, Cai Y, Feng C, Jin F, Jin M. Clinical value of transferring blastocysts derived from 0PN/1PN or grade III embryos. *INTERNATIONAL JOURNAL OF CLINICAL AND EXPERIMENTAL MEDICINE* 2020;**13(9)**:6945-52.

Romanelli V, Fabiani M, Girardi L, Albricci L, Rienzi L, Ubaldi FM, Caroselli S, Cimadomo D, Patassini C, Poli M et al. Parallel analysis of copy number aneuploidies and ploidy level by NGS and SNP array on single biopsy provides accurate results and improves PGT-A applications range. *Human Reproduction* 2019;**34**:135.

Sayers J, Rennison K, Wilson P. The value of atypically fertilised zygotes in an in vitro fertilisation programme. *Human Fertility* 2011;**14(2)**:57-8.

Seckin SI, Jotwani AR, Karipcin SF, Sadowy A, Prosser R, Jalas C, Mounts EL, Hynes JS, Reshef EA, Kappy M et al. EMBRYO RESCUE: EUPLOIDY AND WHOLE CHROMOSOME ANEUPLOIDY RATES IN EMBRYOS INITIALLY CATEGORIZED AS 0 PN AND 1 PN AT FERTILIZATION CHECK. *Fertility and Sterility* 2023;**120(4)**:e64-5.

Shimizu Y, Koi H, Yorimitsu T, Ohara M, Sasaki H, Hasui M, Hondo S, Arichi A, Muramatsu H, Omura N et al. Clinical outcomes of frozen transferred blastocysts derived from monopronucleated (1PN) zygotes after conventional in vitro fertilization (cIVF) and intracytoplasmic sperm injection (ICSI) cycles. *Human Reproduction* 2017;**32**:311-2.

Shun X, Wang J, Huang G, Han W. P-137 pronuclear characteristics and chromosomal constitutions of mono-pronuclear zygotes. *Human Reproduction* 2023;**38(Supplement_1)**: i254.

Si J, Zhu X, Lyu Q, Kuang Y. Obstetrical and neonatal outcomes after transfer of cleavage-stage and blastocyst-stage embryos derived from monopronuclear zygotes: a retrospective cohort study. *Fertility and Sterility* 2019;**112(3)**:527-33.

Soler N, Bautista-Llàcer R, Escrich L, Oller A, Grau N, Tena R, Insua MF, Ferrer P, Escribà MJ, Vendrell X. Rescuing monopronucleated-derived human blastocysts: a model to study chromosomal topography and fingerprinting. *Fertility and Sterility* 2021;**116(2)**:583-96.

Staessen C, Van Steirteghem AC. The chromosomal constitution of embryos developing from abnormally fertilized oocytes after intracytoplasmic sperm injection and conventional in-vitro fertilization. *Human Reproduction* 1997;**12(2)**:321-7.

Stock-Myer S, Kelley R, Agresta F, Gurner K, Williams C, Parris B, Liew I, Demmers K, Cummins L, Lee L, Cattrall F. O-054 Assessing the ploidy status of atypically fertilised embryos–recovery of embryos for potential clinical use. *Human Reproduction* 2025;**40(Supplement_1)**:i34.

Tan K, Zhang SP, Gong F, Xiong B, Lu CF, Lu GX, Lin G. Identification of non-parthenogenetic and euploid blastocysts from one pronuclear oocyte for embryo transfer by single nucleotide polymorphism (SNP) array. *Human Reproduction* 2014;**29**:293.

Tong X, Jin J, Xue Y, Fang L, Zhu H, Jiang L, Zhang S. Clinical outcomes of frozen–thawed blastocysts from zygotes with no or one pronucleus for in vitro fertilization and intracytoplasmic sperm injection cycles. *Archives of Gynecology and Obstetrics* 2023;**308(3)**:1015-22.

Tsuji H, Kitasaka H, Fukunaga N, Asada Y. BIRTH OF HEALTHY BABIES FROM BLASTOCYSTS DERIVED FROM MONO-PRONUCLEAR ZYGOTES FOLLOWING INTRACYTOPLASMIC SPERM INJECTION. *Fertility and Sterility* 2020;**114(3)**:e123-4.

Tsuji H, Tokoro M, Fukunaga N, Asano E, Kounogi S, Asada Y. Clinical utility of monopronuclear zygotes obtained after intracytoplasmic sperm injection (ICSI). *Fertility and Sterility* 2016;**106(3)**:e359-60.

Ussher C, Holden S, Bowman M, McArthur S. O-180 What is the true potential of 1PN embryos? A report on utilisation and live birth rates following PGT-A and biparental testing. *Human Reproduction* 2023;**38(Supplement_1)**:i111.

Wang T, Si J, Wang B, Yin M, Yu W, Jin W, Lyu Q, Long H. Prediction of live birth in vitrified-warmed 1PN-derived blastocyst transfer: Overall quality grade, ICM, TE, and expansion degree. *Frontiers in Physiology* 2022;13:964360.

Wang J, Xie H, Zou Y, Gao M, Wang L, Liu X, Huang SX, Yan J, Gao Y. The use of blastocysts developing from nonpronuclear and monopronuclear zygotes can be considered in PGT-SR: a retrospective cohort study. *BMC Pregnancy and Childbirth* 2025;**25(1)**:530.

Wei YL, Zhu GJ, Ren XL, Huang B, Jin L. Developmental Potential and Clinical Value of Embryos with Abnormal Cleavage Rate. *Current Medical Science* 2019;39(1):118-21.

Wen T, Meng T, Ma Y, Xu Y, Xu Y. PARENTAL INHERITANCE OF MONOPRONUCLEAR BLASTOCYSTS FROM ICSI CYCLES AND ITS CORRELATION WITH PARTICULAR MORPHOKINETIC CHARACTERISTICS. *Fertility and Sterility* 2024;**122(4)**:e198-9.

Werlin LB. Are embryos normal obtained from second-day intracytoplasmic sperm injection (ICSI), single pronucleus (1PN), or cleaved embryos from no pronuclei (0PN) initially seen?. *Fertility and Sterility* 2007;**87(4)**:S11-2.

Wozniak K, Jones A, Kwon M, Zou P, Schiewe MC. CLINICAL BENEFITS OF CULTURING INTRACYTOPLASMIC SPERM INJECTION-DERIVED NONPRONUCLEATED (0PN) AND MONOPRONUCLEATED (1PN) ZYGOTES, AND THE IMPORTANCE OF VERIFYING BIPARENTAL CHROMOSOME INHERITANCE IN 1PN-DERIVED BLASTOCYSTS. *Fertility and Sterility* 2021;**116(3)**:e3.

Wozniak K, Schiewe MC. Clinical benefits of culturing monopronucleated zygotes derived from intracytoplasmic injection cycles. *Reproductive BioMedicine Online* 2020;**41**:e1-2.

Xie PY, Tang Y, Hu L, Ouyang Q, Gu YF, Gong F, Leng LZ, Zhang SP, Xiong B, Lu GX et al. Identification of biparental and diploid blastocysts from monopronuclear zygotes with the use of a single-nucleotide polymorphism array. *Fertility and Sterility* 2018;**110(3)**:545-554.

Xiong S, Wang J, Gao Y, Wu L, Hao X, hong Zhu J, Tong K, Huang G, xia Liu J, Han W. Are the number of nucleolar precursor body and size of pronuclear correlated with embryo development and ploidy status in 1PN zygotes?: an analysis through the time-lapse monitoring and pre-implantation genetic testing. *Zygote* 2024;**32(6)**:459-65.

Xu J, Zhang M, Niu W, Yao G, Sun B, Bao X, Wang L, Du L, Sun Y. Genome-wide uniparental disomy screen in human discarded morphologically abnormal embryos. *Scientific Reports* 2015;**5(1)**:12302.

Xu Q, Mao X, Zhang J, Wu L. Nonpronuclear-and monopronuclear-derived blastocysts do not impair subsequent perinatal and maternal outcomes. *The Journal of Clinical Endocrinology & Metabolism* 2025;**110(4)**:e953-61.

Yao G, Xu J, Xin Z, Niu W, Shi S, Jin H, Song W, Wang E, Yang Q, Chen L. Developmental potential of clinically discarded human embryos and associated chromosomal analysis. *Scientific reports* 2016;**6(1)**:23995.

Yelke HK, Ozkara G, Yildiz S, Colakoglu YK, Aygün TM, Yapan ÇÇ, Kahraman S. USEFULNESS OF CHROMOSOMAL ANALYSIS OF BLASTOCYST DERIVED FROM ABNORMALLY FERTILIZED ZYGOTES INCUBATED IN TIME LAPSE MONITORING (TLM) SYSTEM AFTER ICSI. *Fertility and Sterility* 2023;**120(4)**:e215.

Yeshua A, Cinnioglu C, Jordan A, Devine K, Harris BS, Romanski PA. CHROMOSOMAL ANALYSIS OF BLASTOCYSTS DERIVED FROM 0PN AND 1PN ZYGOTES. *Fertility and Sterility* 2024;**122(1)**:e9-10.

Zhang Y, Hu J, Zhang C, Liu Y, Yang L, Xu Y, Li D, Ren B, Guan Y, Sun L. Effect of frozen-thawed blastocyst transfer of different pronuclear zygotes origins on clinical pregnancy outcome and neonatal outcome. *Chinese Journal of Reproduction and Contraception* 2023;**43(9)**:904-912.

Zhang Y, Zhai Y, Fu L, Li Y. Analysis of pregnancy outcome of embryos derived from abnormal prokaryotes. *Zhonghua yi xue za zhi* 2019;**99(29)**:2308-10.

Zhao H, Yuan P, Chen X, Lin H, Zhao J, Huang J, Qiu Q, Ji X, Zhang Q, Wang W. The aneuploidy testing of blastocysts developing from 0PN and 1PN zygotes in conventional IVF through TE-biopsy PGT-A and minimally invasive PGT-A. *Frontiers in Reproductive Health* 2022;**4**:966909.

Zhu J, Wang C, Cao Z, Luan K, Wu Y, Yin H. Developmental competence and neonatal outcomes of nonpronuclear zygotes following single vitrified-warmed blastocyst transfers using propensity score matching analysis. *Archives of Gynecology and Obstetrics* 2024;**309(1)**:295-304.

Zhu M, Dong Q, Zhu Y, Le Y, Wang T, Zhou Y, Yang S. Developmental potential of non-and mono-pronuclear zygotes and associated clinical outcomes in IVF cycles. *Frontiers in Endocrinology* 2024;**15**:1361734
